# Cell Cycle-Independent Phospho-Regulation of Fkh2 during Hyphal Growth Regulates *Candida albicans* Pathogenesis

**DOI:** 10.1371/journal.ppat.1004630

**Published:** 2015-01-24

**Authors:** Jamie A. Greig, Ian M. Sudbery, Jonathan P. Richardson, Julian R. Naglik, Yue Wang, Peter E. Sudbery

**Affiliations:** 1 Department of Molecular Biology and Biotechnology, University of Sheffield, Western Bank, Sheffield, United Kingdom; 2 Institute of Molecular and Cell Biology, Agency for Science, Technology and Research, Singapore; 3 Department of Physiology, Anatomy and Genetics, University of Oxford, Oxford, United Kingdom; 4 Mucosal and Salivary Biology Division, King’s College London Dental Institute, King’s College London, London, United Kingdom; 5 Department of Biochemistry, Yong Loo Ling School of Medicine, National University of Singapore, Singapore; Texas A&M University, UNITED STATES

## Abstract

The opportunistic human fungal pathogen, *Candida albicans*, undergoes morphological and transcriptional adaptation in the switch from commensalism to pathogenicity. Although previous gene-knockout studies have identified many factors involved in this transformation, it remains unclear how these factors are regulated to coordinate the switch. Investigating morphogenetic control by post-translational phosphorylation has generated important regulatory insights into this process, especially focusing on coordinated control by the cyclin-dependent kinase Cdc28. Here we have identified the Fkh2 transcription factor as a regulatory target of both Cdc28 and the cell wall biosynthesis kinase Cbk1, in a role distinct from its conserved function in cell cycle progression. In stationary phase yeast cells 2D gel electrophoresis shows that there is a diverse pool of Fkh2 phospho-isoforms. For a short window on hyphal induction, far before START in the cell cycle, the phosphorylation profile is transformed before reverting to the yeast profile. This transformation does not occur when stationary phase cells are reinoculated into fresh medium supporting yeast growth. Mass spectrometry and mutational analyses identified residues phosphorylated by Cdc28 and Cbk1. Substitution of these residues with non-phosphorylatable alanine altered the yeast phosphorylation profile and abrogated the characteristic transformation to the hyphal profile. Transcript profiling of the phosphorylation site mutant revealed that the hyphal phosphorylation profile is required for the expression of genes involved in pathogenesis, host interaction and biofilm formation. We confirmed that these changes in gene expression resulted in corresponding defects in pathogenic processes. Furthermore, we identified that Fkh2 interacts with the chromatin modifier Pob3 in a phosphorylation-dependent manner, thereby providing a possible mechanism by which the phosphorylation of Fkh2 regulates its specificity. Thus, we have discovered a novel cell cycle-independent phospho-regulatory event that subverts a key component of the cell cycle machinery to a role in the switch from commensalism to pathogenicity.

## Author Summary

The fungus *Candida albicans* is a commensal in the human microbiota, responsible for superficial infections such as oral and vaginal thrush. However, it can become highly virulent, causing life-threatening systemic candidemia in severely immunocompromised patients, including those taking immunosuppressive drugs for transplantation, sufferers of AIDS and neutropenia, and individuals undergoing chemotherapy or at extremes of age. With a rapidly increasing ageing population worldwide, *C. albicans* and other fungal pathogens will become more prevalent, demanding a greater understanding of their pathogenesis for the development of effective therapeutics. Fungal pathogenicity requires a coordinated change in the pattern of gene expression orchestrated by a set of transcription factors. Here we have discovered that a transcription factor, Fkh2, is modified by phosphorylation under the control of the kinases Cdc28 and Cbk1 in response to conditions that activate virulence factor expression. Fkh2 is involved in a wide variety of cellular processes including cell proliferation, but this phosphorylation endows it with a specialized function in promoting the expression of genes required for tissue invasion, biofilm formation, and pathogenesis in the host. This study highlights the role of protein phosphorylation in regulating pathogenesis and furthers our understanding of the pathogenic switch in this important opportunistic fungal pathogen.

## Introduction

The fungus *Candida albicans* is commonly found as a harmless commensal on the skin and mucosal surfaces of the vaginal and gastrointestinal tracts of healthy people. However, it is also an opportunistic pathogen causing diseases that range from superficial infections, such as vaginal and oral thrush in otherwise healthy people, to life-threatening bloodstream infections that disseminate to internal organs in immunocompromised patients [1–3]. A key aspect of *C. albicans* pathogenicity is the capability to grow in both budding yeast and hyphal forms [4, 5]. When growing at low densities on mucosal surfaces *C. albicans* mostly exists as a commensal and is tolerated by the host immune system [6, 7]. Hyphal and pseudohyphal forms are found at sites of mucosal infections and are responsible for tissue invasion and damage [8, 9]. Hyphae preferentially invade epithelial cells, either by active penetration or host-mediated endocytosis [10–13]. Yeast cells in the bloodstream are engulfed by macrophages [14], but immediately switch to hyphal growth to escape and invade internal organs [15]. Hyphal forms are also a key part of the structure of biofilms [16]. Biofilm formation on the surfaces of implant medical devices has been recognized as a primary source of invading fungal cells, because biofilms provide protection against the host immune system and anti-fungal drugs [16].

Associated with the yeast-hyphal morphological switch, transcriptional changes occur resulting in the expression of proteins required for pathogenesis. This hyphal-specific gene set includes genes required for tissue damage, adhesion and invasion [17]. For example, they encode cell wall proteins such as Hyr1, secreted aspartyl proteases (SAPs) that cause tissue damage [18], and adhesins such as Als3 and Hwp1 that promote hyphal endocytosis by epithelial cells [19, 20]. Transcriptional responses on hyphal induction have been well studied, identifying many genes that are commonly up regulated during the yeast-hyphal switch [21–23].

Gene knockout studies have provided invaluable information on the molecular mechanisms underlying the morphological and transcriptional changes involved in *C. albicans* pathogenesis. This has led to the discovery that the cAMP-PKA-Efg1, MAPK-Cph1, and pH-responsive pathways play a key role in transcriptionally activating the hyphal program, along with the identification of several transcriptional repressors such as Nrg1, Tup1 and Sfl1 [5, 24]. Among the many hyphal-specific genes identified so far, only a few are required for hyphal formation. One example is *HGC1*, which encodes a cyclin homologous to the G1 cyclins Cln1 and Cln2 of the budding yeast *Saccharomyces cerevisiae* that partner the cyclin-dependent kinase (CDK) Cdc28 [25]. Cells lacking *HGC1* are severely defective in hyphal morphogenesis under all conditions tested, and in causing infection in animals.

The discovery of the crucial role of Hgc1 and Cdc28 in *C. albicans* hyphal growth has uncovered multiple regulatory mechanisms involved in hyphal morphogenesis. Rga2 is a negative regulator of Cdc42, a Rho GTPase that orchestrates polarized growth processes at the hyphal tip [26]. Phosphorylation of Rga2 by Cdc28-Hgc1 inhibits its tip localization and keeps Cdc42 in the active state [27]. Cdc28-Cln3 regulates endocytic actin patch dynamics by phosphorylating Sla1, which leads to further phosphorylation by Prk1. Upon hyphal induction, Sla1 is rapidly dephosphorylated resulting in enhanced actin patch activity in hyphae [28]. Sec2 is a secretory vesicle-associated guanine-nucleotide-exchange factor (GEF) for the Rab GTPase Sec4. Phosphorylation of Sec2 by Cdc28-Hgc1 is necessary for its localization to the Spitzenkörper and correct hyphal growth [29]. Cdc28-Ccn1 acts in concert with the Gin4 kinase to phosphorylate a pair of serine residues of the septin Cdc11 within a few minutes of hyphal induction [30]. In the absence of this event, polarized growth is lost after the formation of the first septum.

Another kinase required for hyphal growth is the cell wall integrity kinase Cbk1 and its regulatory subunit Mob2. Cbk1 is a member of the evolutionary conserved Large Tumour Suppressor / Nuclear Dbf2 Related (LATS/NDR) superfamily of kinases that are involved in control of cell shape and growth [31]. In *C. albicans* loss of Cbk1 completely abrogates germ tube formation and polarized growth, disturbs cell separation in yeast cells and reduces expression of hyphal specific genes [32, 33]. Defects in polarised growth are seen when its homologue is lost in other fungi [34]. For example, *orb6* mutants in *Schizhosaccharomyces pombe* [35], *cot1* mutants in *Neurospora crassa* [36] and *cbk1* mutants in *S. cerevisiae* [37, 38] all show profound defects in polarised growth. Cbk1 forms part of the regulation of Ace2 activity and cellular morphogenesis (RAM) network of physically interacting proteins including its activating subunit Mob2, the kinase Kic1, scaffolding proteins Tao1 and Hym1, and the RNA binding protein, Ssd1 [39]. As well as polarised growth the RAM network is also required for cell separation [37, 39]. It phosphorylates the transcription factor Ace2, which then translocates to daughter cell nuclei and transcribes genes that encode hydrolytic enzymes to degrade the primary septum. In *C. albicans* the mechanism of Cbk1 action in hyphal growth and the target proteins it phosphorylates remains largely unknown. One known target of Cbk1 is the transcription factor Bcr1, phosphorylation of which promotes biofilm formation [40].

Clearly, protein phosphorylation plays a key role in morphogenesis and pathogenesis in *C. albicans*. To further investigate the role of phospho-regulation in hyphal growth, we searched for proteins that showed a hyphal-specific pattern of phosphorylation. We observed that the fork-head family transcription factor Fkh2 changes its phosphorylation profile dramatically within five minutes of hyphal induction. In *S. cerevisiae*, two fork-head transcription factors, Fkh1 and Fkh2, control a G2 transcription program including the expression of the G2 cyclin Clb2 required for mitotic entry [41], and the transcription factors Swi5 and Ace2 required for the M to G1 phase gene expression program. We show here that upon hyphal induction in *C. albicans* Fkh2 undergoes a radical shift in its phosphorylation profile mediated by two kinases: Cdc28-Ccn1/Cln3 and Cbk1-Mob2. This shift specifically activates Fkh2 to promote the expression of genes required for pathogenic processes in addition to its normal general housekeeping function. Thus, phosphorylation not only plays a part in promoting polarized growth but also has a role in a novel regulatory circuit that activates hyphal-specific gene transcription necessary for pathogenesis.

## Results

### The phosphorylation profile of Fkh2 changes within five minutes of hyphal induction

**Fig 1:**
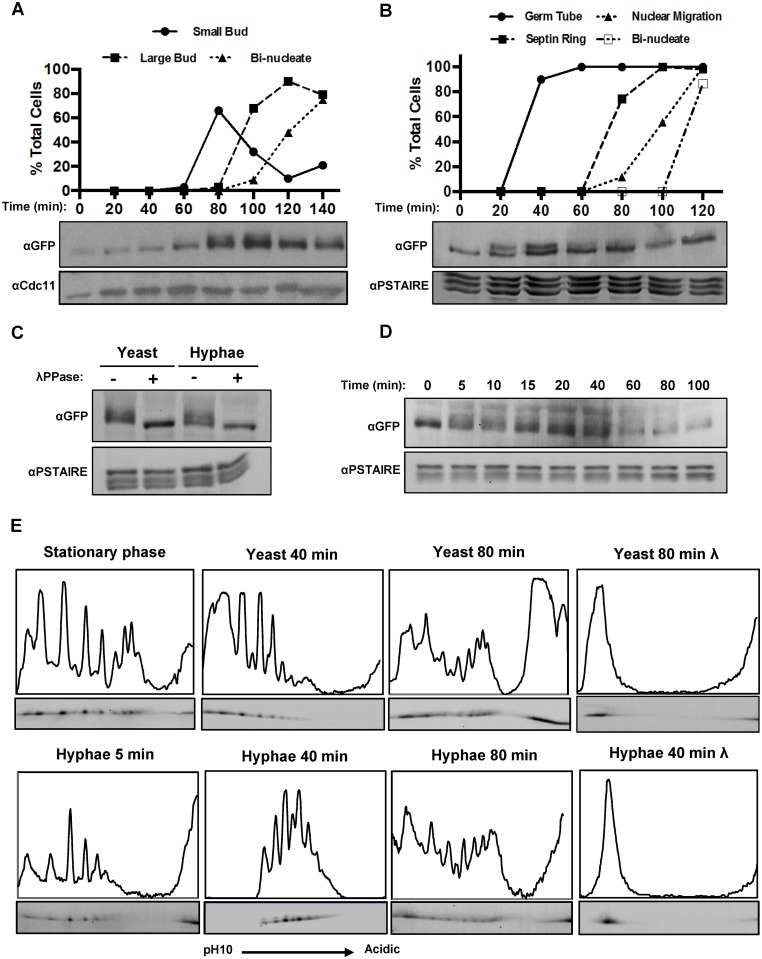
Fkh2 is differentially phosphorylated between yeast and hyphal growth. A) Early G1 cells expressing Fkh2-YFP were collected by elutriation and re-inoculated into yeast growth conditions. Samples were taken for αGFP Western blot to observe Fkh2 phosphorylation and microscopy to follow cell cycle progression via budding and DAPI stained nuclei (n = 50) Note YFP is recognised by the αGFP monoclonal antibody; αCdc11 was used as a control for equal loading. B) Early G1 cells expressing Fkh2-YFP and Cdc12-mCherry were collected by elutriation and re-inoculated into hyphal growth conditions. Samples were taken as above, with cell cycle progression followed by monitoring septin ring formation and nuclear migration/division (n = 50). C) Confirmation of Fkh2 phosphorylation by phosphatase treatment. 80 min yeast and 40 min hyphae samples were taken and lysates treated at 30°C for 1 h with/without Lambda-phosphatase (NEB) and then resolved by 7% 1D PAGE. D) Fkh2 phosphorylation early on hyphal induction. Samples were taken at the indicated time points after hyphal induction and resolved by 1D PAGE as previously mentioned. In Figs. 1B–D αPSTAIRE was used as the loading control. E) Fkh2-YFP was isolated from cells in the culture conditions and times indicated and fractionated by 2D gel electrophoresis. Note the region of darkening at the acidic edge of the gel is where the sample was applied and does not come from Fkh2. An intensity profile is shown above each autoradiograph. In this and subsequent figures the profile was scaled to give maximum height to the maximum peak in the informative part of the gel. Where necessary some values from the non-specific part of the gel were omitted. Fig. 1E is shown with an independent replicate in S2 Fig.

To identify further targets of Cdc28 in hyphal development, we first identified *C. albicans* proteins which contain a cluster of the consensus Cdc28 target motifs, S/TPxK/R (x, any amino acid). We then used a band shift assay in one dimensional polyacrylamide gel electrophoresis (1D PAGE) to determine whether any of these proteins were differentially phosphorylated in hyphae compared to yeast. In addition to the proteins described in the introduction we identified changes in the phosphorylation profile of Orf19.3469 (S1 Fig.), a possible homolog of the *S. cerevisiae* Stb1 protein that regulates the MBF transcription at START [42], Orf19.1948 (S1 Fig.), a protein of unknown function, and Fkh2 which is known to play a key role in cell cycle progression (S1 Fig.). Here we report our analysis of Fkh2 phosphorylation and its cell-cycle independent role in promoting the expression of genes involved in pathogenesis. Fig. 1 presents an experiment where early G1 yeast cells expressing Fkh2-YFP were collected by elutriation and then reinoculated either into yeast growth conditions (YEPD and 30°C, pH 4.0) or hyphal growth conditions (YEPD plus 10% serum and 37°C, pH 7.0). In yeast growth conditions, the appearance of small buds, large buds and binucleate cells was recorded and plotted against time (Fig. 1A). In hyphal cells we plotted germ tube emergence, the appearance of a septin ring within the germ tube as visualized by Cdc12-mCherry fluorescence, nuclear migration as visualised by DAPI staining, and the appearance of binucleate cells (Fig. 1B). A change in the phosphorylation profile of Fkh2 was indicated by the appearance of a double band and the disappearance of the slower migrating band upon phosphatase treatment (Fig. 1C). (Note in Fig. 1A the septin Cdc11 was used as a loading control whereas in Fig. 1B the loading control was Cdc28/Pho85 identified by a monoclonal anti-PSTAIRE antibody). In yeast cells, the Fkh2-YFP band became double after the appearance of small buds (Fig. 1A), consistent with phosphorylation in S-phase as previously documented in *S. cerevisiae* [43]; this then collapsed to one band when the cells became bi-nucleate. In contrast, Fkh2 was present as a double band from 20–60 min after hyphal induction, well before the appearance of the septin ring (Fig. 1B), which marks the start of the cell cycle [44]. Cdc28, partnered by the cyclin Ccn1, and in conjunction with the Gin4 kinase, has been shown to phosphorylate the septin Cdc11 within 5 min of hyphal induction [30]. To determine if Fkh2 is similarly targeted at this early stage, we repeated the experiment collecting samples at 5-min intervals after hyphal induction. Fkh2 showed an additional retarded band after 5 min (Fig. 1D). Thus, whereas Fkh2 is phosphorylated in S-phase in yeast cells, it is rapidly phosphorylated upon hyphal induction in a cell cycle-independent fashion.

In order to generate a more detailed picture of Fkh2 phosphorylation, we carried out two-dimensional (2D) protein electrophoresis using an immobilised pH gradient (IPG) of 3–10 for isoelectric focussing. Quantitative intensity profiles were generated and are displayed above each of the resulting autoradiograms (Fig. 1E). On phosphatase treatment Fkh2 is only present as a single spot at the basic end of the IPG, representing Fkh2 without any negative charges added due to phosphorylation. In contrast to the single band observed in 1D gels in stationary phase, these data showed that there is a diverse pool of differentially charged Fkh2 phospho-isoforms. Five minutes after hyphal induction this profile begins to change and after 40 min of hyphal growth this profile is transformed, but after 80 min it resembles cells growing in the yeast form. A similar change is not observed in cells growing in the yeast morphology 40 min after reinoculation of the stationary phase culture. S2 Fig. shows an independent replicate of this experiment to demonstrate the reproducibility of this key observation. Other 2D gels described in this paper show a similar degree of reproducibility. Thus, shortly after hyphal induction there is a window in which the spectrum of Fkh2 phospho-isoforms is transformed before resuming the characteristic yeast profile.

### Fkh2 is phosphorylated by Cdc28

**Fig 2:**
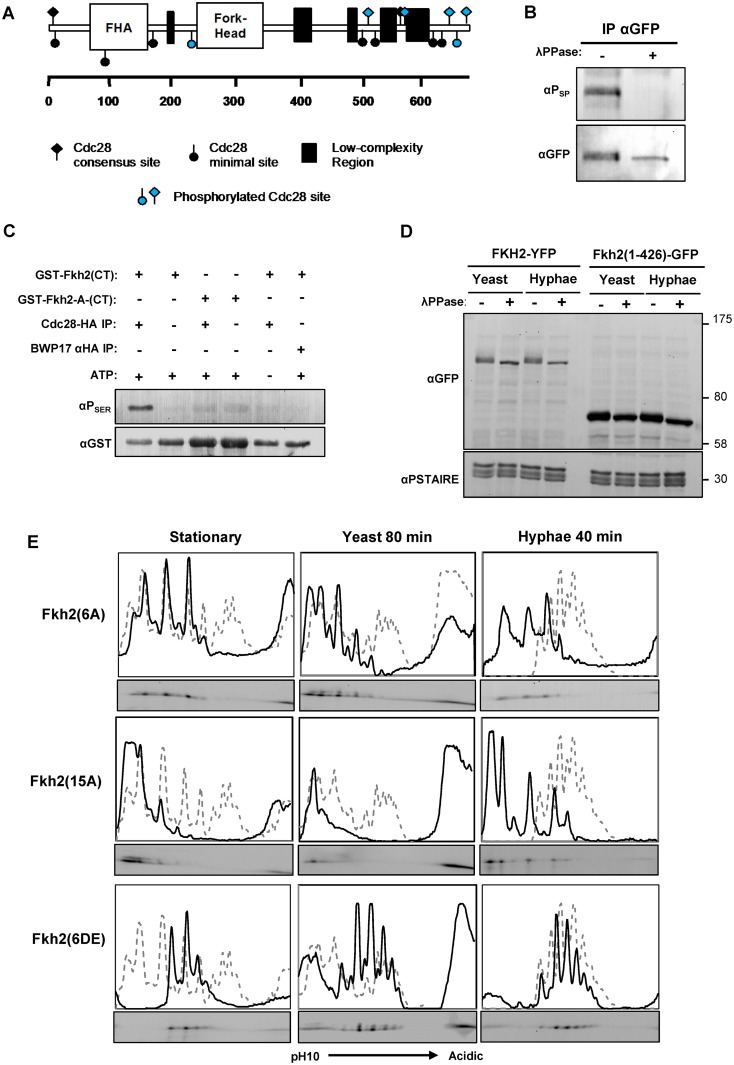
Fkh2 is phosphorylated by Cdc28. A) Schematic showing Cdc28 minimal (circles) and full (diamond) consensus target sites on Fkh2, with those detected by phospho-peptide mapping to be phosphorylated on hyphal induction indicated in blue. B) Phosphorylation of Fkh2 on hyphal induction detected using an antibody that recognises phosphorylated residues in Cdc28 target sites (αP_SER_(CDK) (Cell-Signalling 2324S). C) *In vitro* kinase assay with Cdc28-HA purified from a hyphal lysate and recombinant GST-Fkh2(CT) (aa419–687 intron removed) and GST-Fkh2-A-CT (as GST-Fkh2(CT) fragment with the serine/threonine in the five C-terminal Cdc28 consensus sites mutated to alanine). GST-Fkh2(CT) but not GST-Fkh2-A-CT is phosphorylated *in vitro* by Cdc28-HA. The parental strain BWP17, in which Cdc28 is not HA-tagged, provided the mock lysate to demonstrate that the activity was not due to a co-purifying kinase. D) Removal of Fkh2’s C-terminus containing the Cdc28 target site cluster abolishes the double band upon hyphal induction. *fkh2(1–426)-GFP* and *fkh2/FKH2-YFP* were grown as yeast or hyphae as previously, samples were treated with/without phosphatase and then resolved by SDS-PAGE. E) Autoradiograms from 2D gels and quantitative intensity profiles of the indicated Fkh2 phosphosite mutants at the indicated times and in the indicated culture conditions. The grey dashed line represents the parental Fkh2-YFP profile grown in the corresponding condition as shown in Fig. 1.

**Fig 3:**
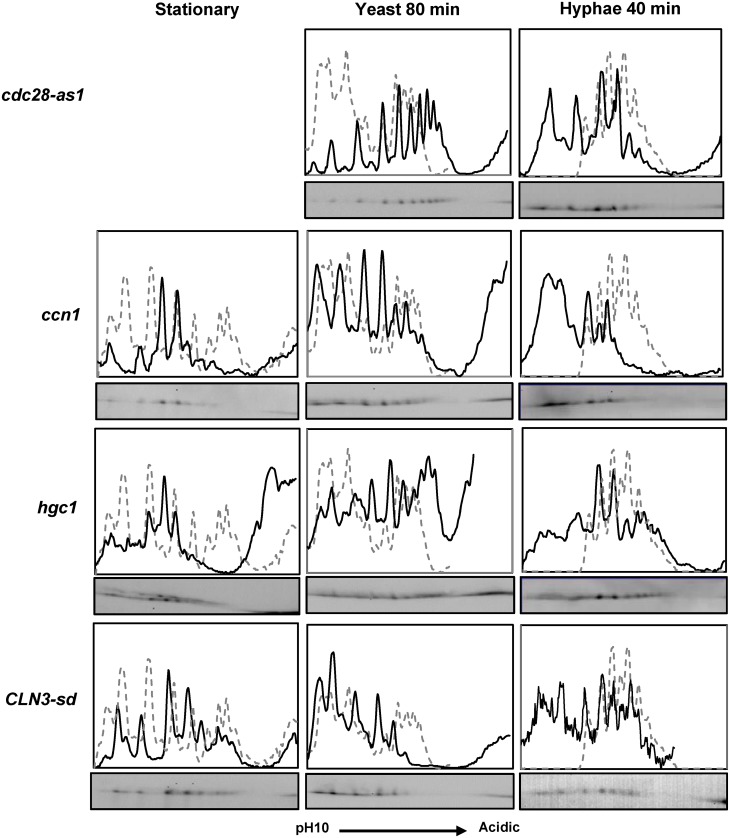
The effect of inhibiting Cdc28 or the removal of Cdc28 cyclins on Fkh2 phosphorylation. Fkh2 from cells of the indicated genotype and culture condition was fractionated by 2D gels. The c*dc28-as1* strain was treated with 30 µM 1NM-PP1 to inhibit Cdc28. Note it is not possible to grow cells to stationary phase with Cdc28 inhibited. The *CLN3-sd* strain was grown to stationary phase overnight in YEPD which allows partial de-repression of the *MET3* promoter regulating *CLN3* expression [76]. For hyphal and yeast growth cells were inoculated from these stationary phase cultures into YEPD medium containing 2.5 mM methionine and 0.5 mM cysteine to repress *CLN3* expression.

To identify the phosphorylated residues on Fkh2 during the window where the shift in the phosphorylation pattern is observed, Fkh2-HA was immuno-precipitated from a hyphal culture 40 min after induction and subjected to phospho-site mapping by mass spectrometry (MS). Six residues were identified with high confidence at four full Cdc28 consensus sites in the C-terminal region and two minimal sites (S/TP), one of which was also in the C-terminal region (Fig. 2A). The full results of the phospho-site mapping are shown in S3 Fig. and S1 Dataset Fkh2-YFP immuno-precipitated from a hyphal culture was detected by an antibody that recognizes phosphorylated serine in the context of a full Cdc28 target sequence (SPxK/R) in a Western blot (Fig. 2B), thus providing further evidence that these Cdc28 target sites are phosphorylated. To test this conclusion more fully and to investigate the physiological role of the phosphorylation, we constructed strains expressing mutant versions of Fkh2 that had the six MS-identified and other potential phospho-acceptor residues replaced by either the non-phosphorylatable alanine (A) or the phosphomimetic glutamate/aspartate (E/D) residues in the following combinations: 1) Fkh2(6AMS) had all six MS-identified sites substituted with alanine; 2) Fkh2(6A) or Fkh2(6DE) carried the indicated substitutions at the six Cdc28 consensus sites including the four C-terminal sites identified by MS and additional two full Cdc28 sites not detected by MS (Fig. 2A); 3) Fkh2(10A) carried the indicated substitutions at all the full and minimal Cdc28 sites C-terminal to the DNA binding domain; 4) Fkh2(15A) or Fkh2(15DE) harboured the indicated substitutions at all the full and minimal Cdc28 sites across the whole protein; and 5) Fkh2(1–426) was a truncated version with the C-terminal domain, containing five full and five minimal Cdc28 sites, removed. In each case, the mutant protein was C-terminally fused to GFP. We confirmed that the Fkh2-GFP protein was functional, because the *fkh2*/*FKH*2-*GFP* strain did not show the *fkh2ΔΔ* phenotype previously described [45] and also shown below in this study. We also confirmed that the mutant proteins were present in similar levels to the wild-type protein (S3D,E Fig.), or in the case of Fkh2(1–426) the protein more abundant than the wild-type protein (Fig. 2D).

To test the hypothesis that Cdc28 directly phosphorylates Fkh2 at these sites, we used an *in vitro* kinase assay to demonstrate that immuno-purified Cdc28-HA can phosphorylate an *E. coli*-expressed recombinant C-terminal GST-Fkh2 fragment (Fig. 2C); however, the C-terminal fragment carrying the serine/threonine to alanine substitutions in the Cdc28 consensus sites was not a substrate as predicted by this hypothesis. We then examined whether these phospho-acceptor substitutions affected Fkh2 phosphorylation *in vivo*. The Fkh2(1–426)-GFP C-terminal truncation did not show the characteristic double band of the wild-type protein, suggesting that phosphorylation of the cluster of C-terminal Cdc28 target sites contribute to the band shift (Fig. 2D). 1D gels of the mutants showed that Fkh2(6AMS) was still phosphorylated, by the presence of a more retarded isoform (S3D Fig.). The Fkh2(6A), Fkh2(10A) and Fkh2(15A) proteins were only present as one phospho-isoform on 1D-PAGE; whereas the Fkh2(6DE) and Fkh2(15DE) proteins still showed a more retarded phospho-isoform (S3E,F Fig.). We used 2D gels to further examine the phosphorylation profile of the above phospho-site mutants in stationary phase, growing yeast cells, and hyphal cells early after induction. The results are shown in Fig. 2E. In each panel the profile of the wild-type Fkh2 protein in the corresponding culture condition is shown as a grey dashed line for comparison. The Fkh2(6A) mutant showed only three major spots in both stationary phase, growing yeast and early hyphal growth in contrast to the more complex pattern of the parental cells (Fig. 2E). Thus, Fkh2 is phosphorylated at these sites in both yeast and hyphae and requires this phosphorylation to show the characteristic early hyphal profile. The Fkh2(15A) mutant, which lacks all 15 possible Cdc28 target sites, shows little, if any evidence of phosphorylation in stationary phase and in yeast growth conditions. However, 40 min after hyphal induction additional peaks are present providing clear evidence of phosphorylation events that are specific to the early period of hyphal induction. Nevertheless, the profile is different from the characteristic early hyphal pattern showing that the Cdc28 sites are required for the transition to the early hyphal profile. These peaks could represent additional cryptic Cdc28 target sites that are specific to the early hyphal form or they could indicate the action of one or more additional kinases. Like the Fkh2(6A) mutant, the Fkh2(6DE) mutant is also present as three spots in stationary phase (Fig. 2E), but they are shifted to the more acidic end of the pH gradient due to the negative charge on the glutamate/aspartate residues. Interestingly, the Fkh2(6DE) mutant is able to undergo further phosphorylation on hyphal induction to generate the hyphal specific profile. This suggests that a second kinase acts on Fkh2 after hyphal induction in addition to phosphorylation at the Cdc28 consensus sites and is consistent with the evidence from the Fkh2(15A) mutant, which is still present as multiple phospho-isoforms even though all the potential Cdk1 target sites are blocked in this mutant.

We next examined the Fkh2 2D profile in the *cdc28-as1* mutant, and in mutants in which one of the Cdc28 G1 cyclins was either deleted (*hgc1ΔΔ* and *ccn1ΔΔ*) or down regulated using the *MET*3 promoter (*CLN3-sd*) (Fig. 3). The results showed that both inhibition of Cdc28 or lack of either Ccn1 or Cln3 prevented the shift to the hyphal profile, but there was less of an effect on the early hyphal profile in cells that lack Hgc1. Thus, the data support the conclusion that Cdc28 acts on Fkh2 early after hyphal induction, but suggest both Cln3 and Ccn1 are required to partner Cdc28. Inhibition of Cdc28 or lack of Cln3 also had a major effect on the Fkh2 profile in growing yeast cells, similar to the effect of the Fkh2(6A) mutant in these cells. Thus, while Cdc28 mediated phosphorylation is necessary for the transition to the early hyphal pattern, Fkh2 is also phosphorylated by Cdc28 during yeast growth, as would be expected from previous studies in *S. cerevisiae*.

### Phosphorylation of Fkh2 programs the transcription of genes required for biofilm formation and host interaction

**Fig 4:**
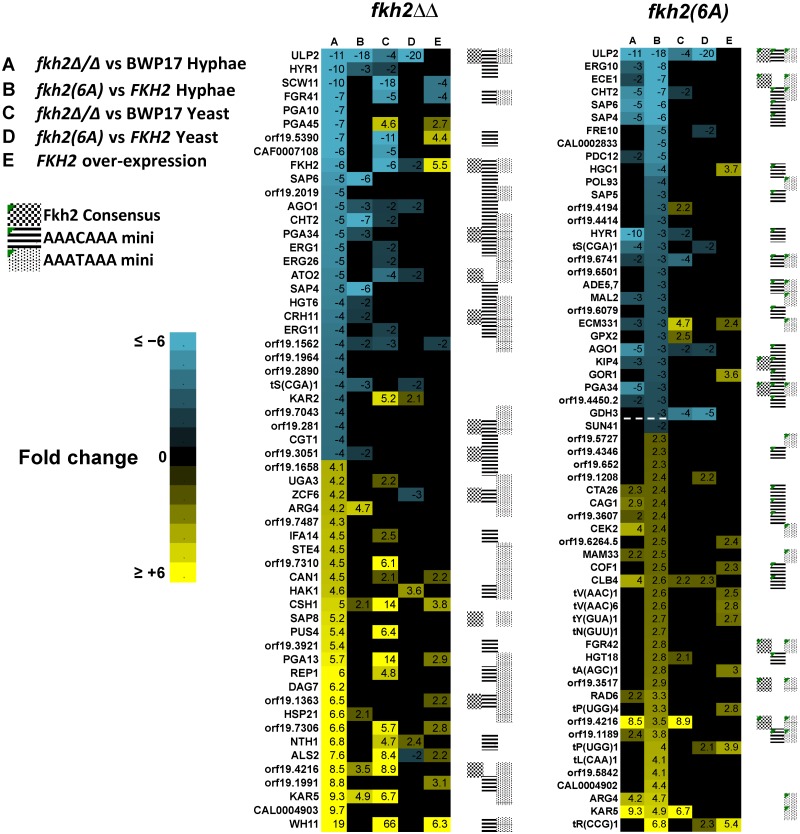
Microarray analysis of Fkh2 mutants. Microarray results sorted for the top 30 genes down/up regulated compared to the parental strain in the *fkh2(6A)* or *fkh2ΔΔ* mutants as indicated. Note the gene ranked 30 down regulated in the *fkh2(6A)* mutant (orf19.715 3-fold down) has been replaced with *SUN41* (2-fold down; adjusted p-value = 5.4 × 10^-5^) as indicated by the dotted line. The full microarray of genes up or down regulated in all Fkh2 mutants is available in S2 Dataset. Gene annotation was provided from the *Candida* genome database http://www.candidagenome.org. Also shown is the presence of a predicted Fkh2 consensus (G/ATAAAC/TAAA) or minimal (AAAT/CAAA) binding site in the upstream 1 kb of each gene.

**Fig 5:**
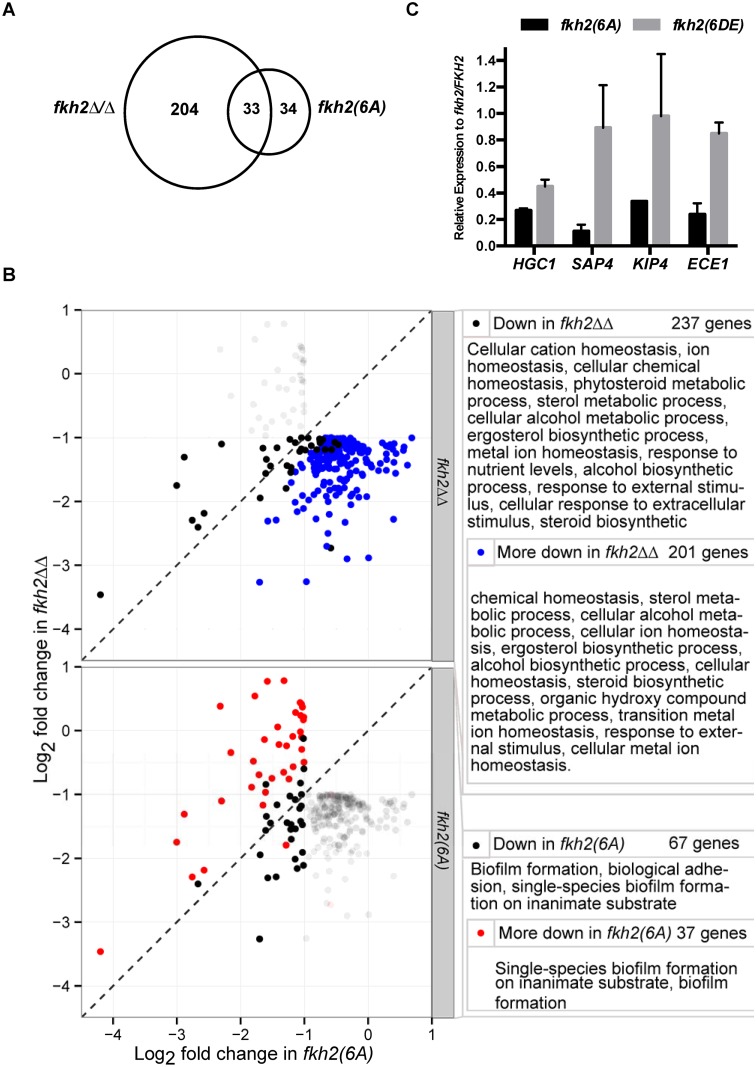
GO analysis of *fkh2(6A)* and *fkh2ΔΔ* mutants. A) Venn diagram showing the number of genes down regulated in the *fkh2(6A)* and *fkh2ΔΔ* mutants and the overlap in the two data sets. B) Genes significantly more down regulated in *fkh2ΔΔ* mutant compared to the *fkh2(6A)* mutant and genes more down regulated in the *fkh2(6A)* mutant compared to *fkh2ΔΔ*. Each panel shows the genes down regulated at least two fold at the 5% FDR threshold in *fkh2(6A)* or *fkh2ΔΔ* (empirical Bayes moderated t-test). Genes significantly down regulated in *fkh2ΔΔ* (above) or *fkh2(6A)* (below) are highlighted in the solid points. Those genes significantly (5% FDR, empirical Bayes moderated t-tests) more down regulated in *fkh2ΔΔ* than *fkh2(6A)* are shown in blue (top panel), or more down regulated in *fkh2(6A)* than *fkh2ΔΔ* are shown in red (lower panel). Right: GO pathways enriched in genes in each category highlighted on the left. Note for reasons explained in the text the figures in this panel are not consistent with the Venn diagram shown in panel A. C) qPCR comparing the expression of hyphal associated transcripts in the Fkh2 phosphorylation mutants. Expression levels are normalised against *ADE2* and shown relative to the expression in the *fkh2/FKH2* strain. Means are from two independent biological repeats, each with three technical replicates. Vertical bars are equal to one standard error.

To investigate the physiological role of Fkh2 phosphorylation after hyphal induction, we used microarrays to compare the transcriptome of the *fkh2(6A)* and *fkh2ΔΔ* mutants with their respective parental strains (*fkh2(6A)-GFP* versus *FKH2-YFP* and *fkh2ΔΔ* versus *FKH2/FKH2*) (Figs. 4, 5 and S2 Dataset). Previous microarray analysis with the *fkh*2*ΔΔ* mutant used a limited array containing probes for only 319 *C. albicans* ORFs, thus likely under-representing the transcriptional role of Fkh2 [45]. To provide a more complete analysis of the role of Fkh2, we also carried out microarray analysis using the above strains in exponentially growing yeast cultures as well as hyphal cultures. We also examined the effect of over-expressing *FKH2* driven by the *GAL1* promoter in yeast cultures, since overexpression of *FKH2* has been shown to induce hyphal-like growth [46]. Full microarray results can be found in S2 Dataset.

Many genes known to be up regulated during hyphal growth and pathogenesis were found to be down-regulated in both the *fkh2ΔΔ* and *fkh2(6A)* mutants (Fig. 4). These included *HYR1* and *ECE1*, which have been previously shown to be down-regulated in the *fkh2ΔΔ* strain [45]. In addition, the secreted aspartyl protease genes *SAP4* and *SAP6*, the chitinase gene *CHT*2, and the hyphal-specific kinesin-like protein gene *KIP4* were also down-regulated in both the *fkh2(6A)* and *fkh2ΔΔ* mutants (Fig. 4). The overlap between the sets of genes down regulated in the two strains is summarised by the Venn diagram in Fig. 5A. However, comparing the gene sets in this way is potentially misleading for two reasons. First, while the degree of down-regulation may be greater than an arbitrary threshold in both mutant strains, the degree of down-regulation may actually be significantly different: e.g. 2.1-fold down in one strain compared to 10-fold down in the second strain. Second, the degree of down-regulation may actually be quite similar, but exceeds the arbitrary threshold in one strain but just fails to exceed the threshold in the second strain: e.g. 1.9-fold compared to 2.1-fold down-regulated. For this reason we compared the fold change for every gene in the *fkh2(6A)* and *fkh2ΔΔ* data sets to determine which genes showed a significantly greater degree of down regulation in each of the two mutants (S2 Dataset). The results are summarised in Fig. 5B together with the Gene Ontogeny (GO) processes co-ordinately affected in each mutant together with the GO processes of those genes that were significantly more affected in one mutant compared to the other.

Of the 237 genes down-regulated in *fkh2ΔΔ*, the change was significantly greater in *fkh2ΔΔ* than in *fkh2(6A)* cells in 201 cases (FDR threshold 0.05, empirical Bayes moderated t-statistic). Of the 67 genes down-regulated in the *fkh2(6A)* mutant, the change was significantly greater in this mutant than in *fkh2ΔΔ* in 37 cases (FDR threshold 0.05, empirical Bayes moderated t-statistic). GO analysis showed that the genes only affected in the *fkh2ΔΔ* strain during hyphal growth were associated with a wide range of metabolic processes including sterol and ergosterol biosynthesis, ion homeostasis and filamentous growth (Fig. 5B). In contrast, GO analysis showed that genes down-regulated in the *fkh2(6A)* mutant were involved in only biofilm formation and biological adhesion (Fig. 5B). Importantly, *HGC1*, the Cdc28 cyclin essential for hyphal development [25], and *SUN41*, which is critically required for hyphal and biofilm formation [47], are only down-regulated in the *fkh2(6A)* mutant (Fig. 4 and S2 Dataset). We confirmed the down-regulation of *HGC1*, *SAP4*, *KIP4* and *ECE1* in the *fkh2(6A)* mutant by qPCR (Fig. 5C). We also showed that in the *fkh2(6DE)* mutant the expression levels of these genes were near wild-type levels with the exception of *HGC1*, whose expression also appears reduced, but not to the extent as seen in the *fkh2(6A)* strain. Thus, correct phospho-regulation of Fkh2 at the six Cdc28 consensus sites is required to specifically activate a subset of genes that are associated with interaction with the host.

There was also a limited overlap between the sets of genes up-regulated during hyphal growth in the *fkh2ΔΔ* mutant compared to the *fkh2(6A)* mutant. However, the GO analysis indicated fewer processes being co-ordinately affected. In the *fkh2ΔΔ* mutant the genes up-regulated were involved in GO processes: oxidative reduction and cellular response to oxidative stress. There were no GO processes that were significantly over-represented in the set of genes up-regulated in the *fkh2(6A)* mutant during hyphal growth. However, *PDE1*, *CLB4* and *RAD6* were all up-regulated in both the *fkh2(6A)* and the *fkh*2*ΔΔ* mutants. *PDE1* encodes a phosphodiesterase that hydrolyses cAMP, thus inhibiting the major signalling pathway for hyphal morphogenesis. Over-expression of *CLB4* may promote the non-polar growth that is characteristic of G2 cyclin mutants. *RAD6* is known to be a negative regulator of hyphal growth [48]. The effect of the *fkh2ΔΔ* mutation was again greater than the *fkh2(6A)* allele during yeast growth. In the *fkh2ΔΔ* cells 125 genes were down-regulated and 219 genes up-regulated. In contrast, in *fkh2(6A)* cells only 37 genes were down-regulated and 42 genes up-regulated. Only 14 genes were down-regulated and 13 genes up-regulated in both cell types (S2 Dataset). There were 24 genes that were down-regulated only in the *fkh2(6A)* mutant. However, there were no GO processes co-ordinately affected in this gene set.

Overexpression of *FKH2* from the *GAL1* promoter resulted in a filamentous phenotype as previously reported [46] (S4 Fig.). Close inspection suggested that these were not true hyphae, but rather resembled the phenotype of cells blocked in the cell cycle by treatments such as hydroxyurea. DAPI staining revealed that elongated daughter cells were often anucleate or contained fragmented nuclei. Microarray analysis revealed down-regulation of the *CDC14* phosphatase and the Gin4 kinase both of which are required for cell cycle progression (S2 Dataset). Furthermore, *DAM1* and *ASK1* were also down-regulated. These genes encode a complex that is required for the coupling of kinetochores to microtubules. Thus, down-regulation of these genes required for progress through mitosis provides a plausible explanation for the filamentous phenotype observed upon *FKH2* overexpression.

### *fkh2(6A)* mutants are defective in hyphal maintenance, substrate invasion, biofilm formation, tissue damage and activation of host immune response

**Fig 6:**
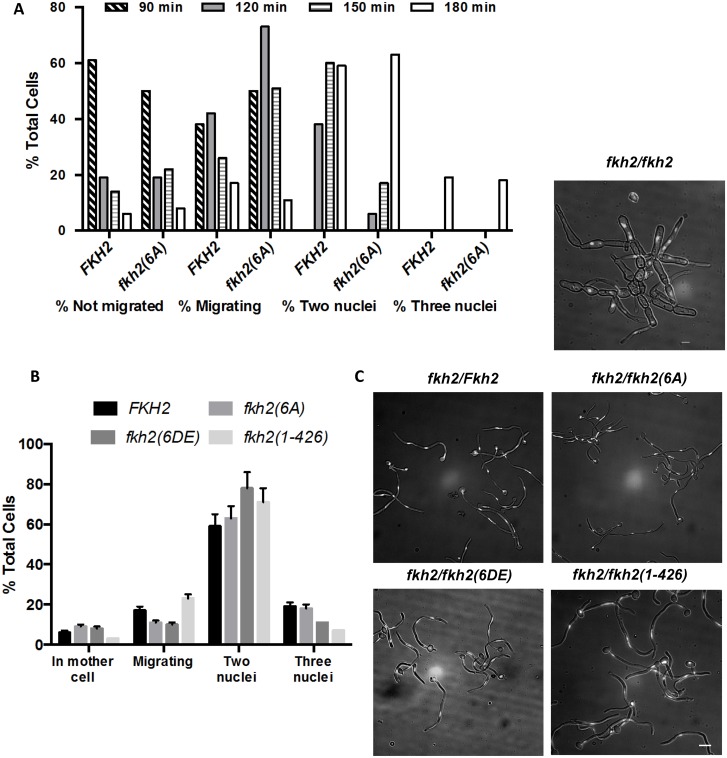
The effect of C-terminal deletion or phosphosite mutations on cell cycle progress during hyphal growth. A) Stationary phase *fkh2/FKH2* and *fkh2(6A)* cells (with a single nucleus) were inoculated into hyphal growth conditions. Samples were taken at 30 min intervals from 90 to 180 min, the hyphal cells were fixed with 1.5% formaldehyde and nuclei stained with DAPI. Hyphae were scored according to whether they had a single nucleus in the mother cell, a single migrating nucleus, two or three nuclei. A minimum of 100 cells were counted. B) Nuclear distribution of the indicated genotypes 180 min after hyphal induction. A minimum of 100 cells were counted. The distribution of nuclei in the *fkh2∆∆* strain was so perturbed that it was not possible to make a meaningful assessment of nuclear content. C) Appearance of cells 180 min after hyphal induction. Images are shown as the merged DAPI (white) and DIC channels, and scale bars are equal to 10 μm. The DIC channel has been darkened for ease of nuclear visualisation.

One explanation for the altered pattern of gene expression in the *fkh2(6A)* mutant is that this mutant is perturbed in cell cycle progression in hyphae. To address this possibility we quantified the nuclear distribution in *fkh2(6A)* and parental BWP17 cells at 30-min intervals after stationary phase yeast cells were inoculated into hyphal inducing conditions. To do this, we characterised the developing hyphae according to whether they contained a single nucleus in the mother cell, a single nucleus that was in the process of migrating into the developing germ tube, two nuclei thus having finished the first mitosis or three nuclei thus having completed the second mitosis (after the first mitosis the nucleus that returns to the mother cell does not re-enter the cell cycle [49]) (Fig. 6). Nuclear migration commenced 90 min after induction in both wild-type and mutant cells (Fig. 6A). *fkh2(6A)* cells did show a delay in completing the first mitosis (Fig. 6A). However, by 180 min, when the samples for microarray analysis were harvested, *fkh2(6A)* cells had completed the first mitosis and the nuclear distribution of these cells was essentially identical to parental *fkh2/FKH2* cells; indeed approximately 20% of both parental and mutant cells had completed the second mitosis (Fig. 6A–C). Fig. 6B,C also shows the cell cycle distribution of *fkh2(6DE)*, *fkh2(1–426)* and *fkh2ΔΔ* mutants 180 min after hyphal induction. The *fkh2(6DE)* and *fkh2*(1–426) mutants also showed a similar distribution to parental cells except that fewer of these cells had completed the second mitosis. In contrast, the *fkh2ΔΔ* mutant cells failed to form normal hyphae. The cells were swollen and the nuclear distribution was grossly abnormal with some cells containing two nuclei and other cells containing no nuclei (Fig. 6C). Thus, while Fkh2 is essential for normal cell cycle progression, mutations affecting the C-terminal domain have only a mild effect.

**Fig 7:**
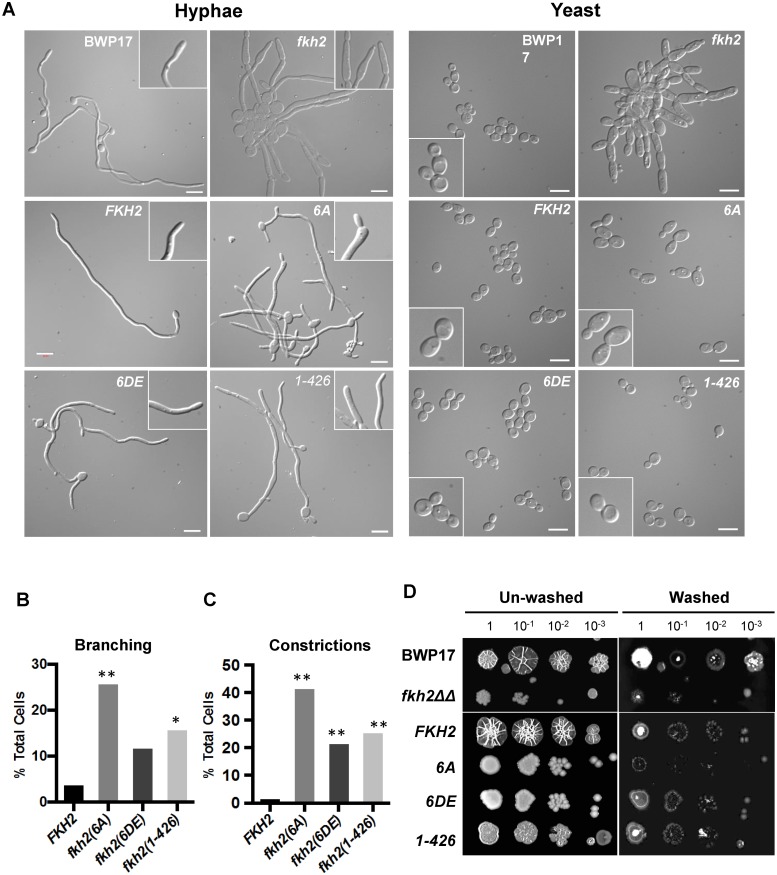
Phosphorylation of Fkh2 affects the long-term maintenance of hyphal growth. A) Phenotypes of Fkh2 phosphorylation mutants of the indicated genotypes. Left: grown as hyphae for 6 h in GMM with 10% FCS, fixed with 1.5% formaldehyde and pepsin treated. White arrows indicate branching and black arrows indicate constrictions. Right: yeast cells were re-inoculated into GMM pH 4.0 for 4hrs before formaldehyde fixation. Scale bars are equal to 10 μm. B) and C). Quantitation (n = 50) of the hyphal phenotypes was carried out to quantitate hyphal branching (B), and constrictions within a hypha (C). For Figs. B and C, a Z-test was used to compare the proportion of cells displaying the phenotype in the mutants with the wild type. * z < 0.05, ** z < 0.01. D) Long-term effects on hyphal growth observed on solid Spider medium. An overnight culture was diluted to OD_600_ = 1.0 and then serially diluted 10 fold as shown, with 1 μl of each dilution being spotted and the plates left at 37°C for five days. The extent of invasion was observed through washing off the surface colony using deionised water.

Microarray analysis revealed that the *fkh2(6A)* mutant is defective in the expression of genes that have been associated with pathogenic processes and host interaction. We therefore went on to characterise the phenotype of the *fkh2(6A)*, *fkh2(6DE)* and *fkh2(1–426)* mutants, to investigate whether they are defective in the corresponding pathogenic processes. The *fkh2(6A)* mutant initially formed hyphae normally as shown by the images after 180 min (Fig. 6C); however, after 6 h of hyphal induction in liquid culture hyphae displayed an increased branching frequency and subtle morphological abnormalities such as swelling of the hyphal tip, not seen in the wild-type cells (Fig. 7A–C). These abnormalities were reduced in the phosphomimetic *fkh2(6DE)* mutant (Fig. 7A–C). However, the *fkhΔΔ* mutant showed pseudohyphal-like growth in yeast growth conditions and more severe hyphal defects than the *fkh2(6A)* mutant (Fig. 7A). On solid Spider medium, both the *fkh2(6A)* and *fkh2(6DE)* mutants failed to produce the wrinkled colony morphology normally seen in the wild-type cells, while the *fkh2(1–426)* mutant showed reduced wrinkling (Fig. 7D). Importantly, the *fkh2(6A)* mutant was defective in invasion of the agar substratum in a wash-off test, whereas the *fkh2(6DE)* and *fkh2(1–426)* strains showed a similar invasive capacity to the *fkh2/FKH2* strain (Fig. 7D). The *fkh2ΔΔ* strain grew poorly on Spider medium and failed to invade the agar. Taken together, these observations suggest that the correct phospho-regulation of Fkh2 is required for long-term hyphal maintenance and invasive growth.

**Fig 8:**
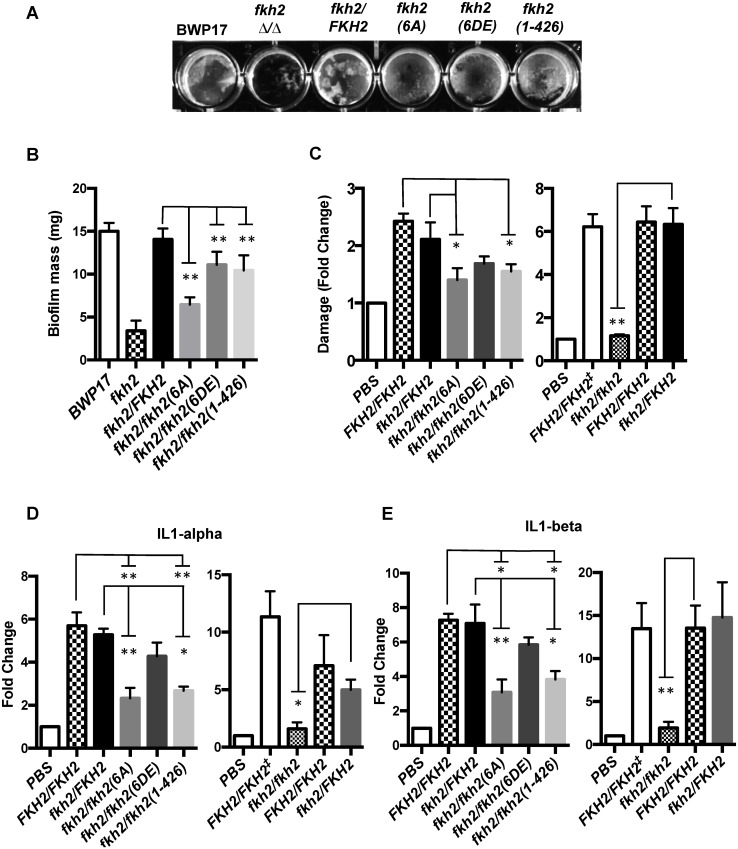
Biological confirmation of the microarray results—phosphorylation of Fkh2 affects multiple pathogenic processes. A) Biofilm formation in 12 well plastic plates imaged after 48 h. B) Quantitation shows the average weight for ten individual biofilms. C) Epithelial cell damage measured by LDH release in a TR146 oral epithelial monolayer infection model. D) and E) Immune activation in the infection model was measured by IL-1α (D) or IL-1β (E) production. Error bars are standard deviations. In C-E *FKH2/FKH2^‡^* represents an *ARG4^+^ URA3^+^* BWP17 strain that has identical auxotrophic requirements to the other strains tested; *FKH2/FKH2* represents a strain with 2 wild-type alleles of *FKH2*, one of which is C-terminally tagged with YFP and is prototrophic for *URA3* only. B-E: Significance of the indicated comparisons was assessed by unpaired, one-tailed t-tests * p ≤ 0.05, ** p ≤ 0.01.

The microarray analysis of the *fkh2(6A)* mutant showed defects in the induction of genes involved in biofilm formation, interaction with host cells, and activation of host immune response. *Fkh2ΔΔ* cells were unable to form biofilms (Fig. 8A,B). The biofilm matrix was visibly reduced in the *fkh2(6A)* strain and the average biofilm mass was only half that of the *fkh2/FKH2* and BWP17 parental strains (Fig. 8A,B). The *fkh2(6DE)* and *fkh2(1–426)* also showed biofilm formation defects, but these were not as severe as those observed in the *fkh2(6A)* strain (Fig. 8A,B). Furthermore, the *fkh2(6A)* mutant was markedly defective in causing tissue damage as measured by a reduction in lactate dehydrogenase release from damaged cells in a TR146 oral epithelial monolayer infection model (Fig. 8C). Cells lacking Fkh2 were completely unable to cause damage, as would be expected from the deleterious phenotype. There was also a significant reduction in the levels of the interleukins IL1-α and IL-1β released when *fkh2(6A)* was used in the infection model (Fig. 8D–E). This suggests that in the absence of Fkh2 phosphorylation *C. albicans* elicits a reduced immune response. The above data show that the changes in gene expression resulting from loss of Fkh2 phosphorylation do indeed have corresponding effects on pathogenic processes. Thus, the change in Fkh2 phosphorylation upon hyphal induction is required to positively regulate multiple virulence mechanisms.

### Phosphorylation of Fkh2 is required to interact with the chromatin modifier Pob3

**Fig 9:**
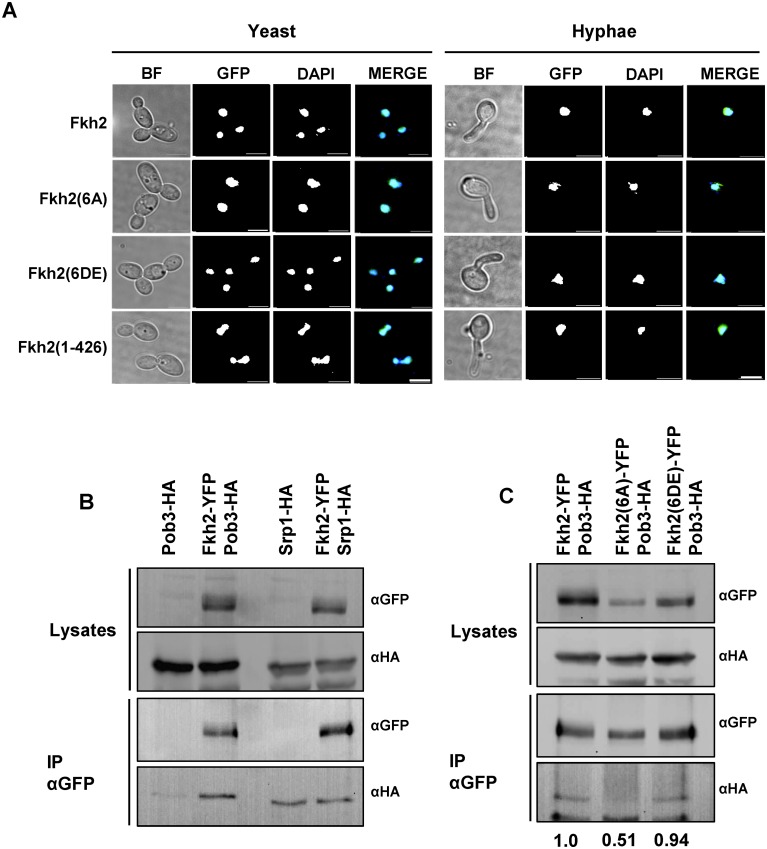
Phosphorylation of Fkh2 does not alter its localisation, but affects its interaction with the chromatin modifier Pob3. A) Fkh2-YFP/GFP localisation in log phase yeast cells or 40 min after hyphal induction. Images are taken at x100 magnification. Scale bar, 5 μm. B) Co-IP experiments of Fkh2 with Pob3 and Srp1 from samples taken 40 min post hyphal induction. 2 mg of total protein was used for IP and 50 μg for the lysates. IP products were washed twice with lysis buffer containing 150 mM NaCl. C) Interaction of Pob3 with Fkh2 phospho-mutants, performed as above. The co-immunoprecipitation was quantified by normalising the αHA signal (Pob3) against the immunoprecipitated αGFP (Fkh2) for each IP, and then expressing this value as a fraction of the wild type value.

To further investigate the physiological role of Fkh2 phosphorylation, we first examined whether it affected Fkh2 nuclear localisation and found that the Fkh2(6A), Fkh2(6DE) and Fkh2(1–426) mutant proteins all localised to the nucleus in the same way as the wild-type protein (Fig. 9A). Next we investigated which proteins interacted with Fkh2 early upon hyphal induction and whether any such interactions were altered by Fkh2 phosphorylation. To do this, Fkh2 was immuno-precipitated from a strain expressing Fkh2–6Myc and fractionated by SDS-PAGE; the proteins revealed by Coommassie Blue staining were then identified by MS. Most of the bands were found to be proteins already present in our database of common contaminants, but one band was identified as a mixture of the *Candida* orthologues of *Sc*Srp1 and *Sc*Pob3. *Sc*Srp1 is a karyopherin [50], while *Sc*Pob3 is a member of the facilitates chromatin transcription (FACT) nucleosome remodelling complex [51]. To verify this finding, we constructed strains co-expressing Pob3-HA or Srp1-HA with Fkh2-YFP and determined whether the proteins could co-immunoprecipitate. Western blot analysis showed that when Fkh2-YFP was immuno-precipitated, a more intense band corresponding to Pob3-HA can be detected in a strain co-expressing Fkh2-YFP and Pob3-HA, than in a strain only expressing Pob3-HA (Fig. 9B). A band corresponding to Srp1-HA could be detected at the same intensity in a strain only expressing Srp1-HA as well as in the co-expressing strain (Fig. 9B), suggesting the Fkh2-Srp1 interaction may be non-specific. Further investigation of the Fkh2-Pob3 interaction showed that when Fkh2(6A)-GFP was immuno-precipitated the amount of Pob3-HA co-precipitated was reduced by half in a co-expressing strain (Fig. 9C). However, the co-immunoprecipitation of Pob3 was present when Fkh2(6DE)-GFP was used as the bait, suggesting that the interaction of Pob3 with Fkh2 is phosphorylation dependent. Thus we have uncovered a possible mechanism through which the phosphorylation of Fkh2 on hyphal induction could bring about the changes in gene expression required for virulence, through mediating the interaction of Fkh2 with Pob3.

### Fkh2 is targeted by Cbk1

**Fig 10:**
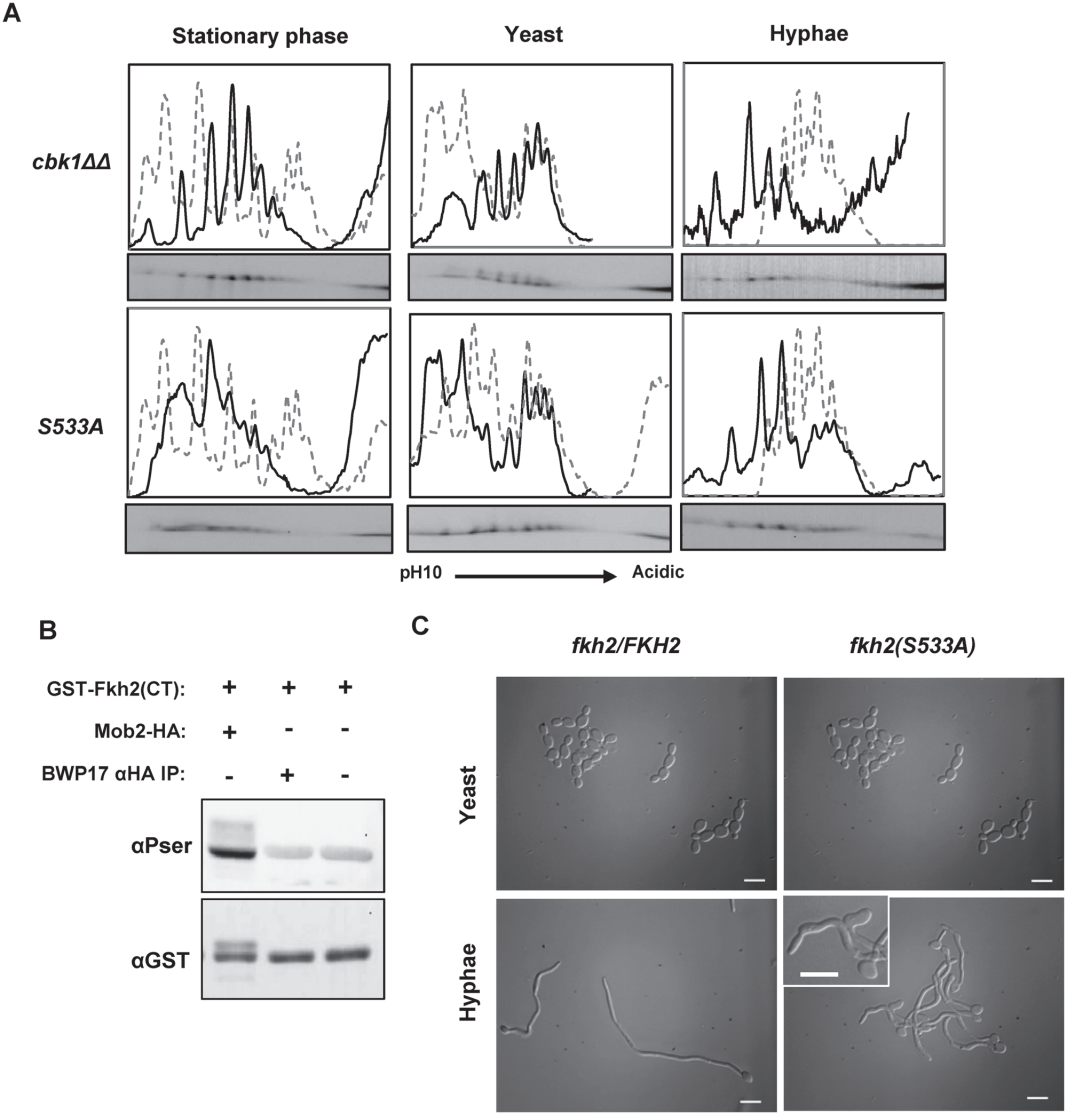
Fkh2 is phosphorylated at serine 533 by Cbk1-Mob2. A) Autoradiograms from 2D gels and quantitative intensity profiles of the indicated strains at the indicated times and culture conditions. The grey dashed line represents the wild type Fkh2-YFP profile grown in the corresponding condition as shown in Fig. 1. B) *In vitro* kinase assay with Mob2-HA purified from a 40 min hyphal culture and *E. coli* expressed Fkh2 C-terminal fragment used in Fig. 2C. A BWP17 mock lysate controls for the possibility of a co-precipitating kinase from the αHA IP. C) Morphology of *fkh2* S533A mutants. *fkh2/FKH2* and *fkh2/fkh2(S533A)*. Cells were re-inoculated from stationary phase cultures into either yeast or hyphal growth conditions, grown for 6 h and then fixed with 1.5% formaldehyde. Hyphal cells were pepsin treated to remove clumps before imaging at x100 magnification. Scale bars represent 10 μm.

Residual phosphorylation was observed in the Fkh2(15A) and Fkh2(6DE) mutants during early hyphal growth and in *cdc28-as1* cells upon the addition of inhibitor (Fig. 2E). This suggests that an additional kinase may be targeting Fkh2. We used 1D gels to profile Fkh2-GFP in a number of kinase mutants and found an altered profile in cells lacking the Cbk1 kinase (S5 Fig.). We therefore used 2D gels to profile Fkh2 in *cbk1ΔΔ* cells and found that Fkh2 failed to show the characteristic early hyphal profile (Fig. 10A). Interestingly, although the stationary phase profile was also altered, the profile of growing yeast was largely unaltered. *S. cerevisiae* Cbk1 has been shown to preferentially target serine or threonine with histidine at-5, arginine at-3 and serine at-2 [52]. Such a site is present in the C-terminal domain of *Ca*Fkh2 (528-HSRSTS-533). We constructed a strain expressing Fkh2 with a non-phosphorylatable substitution at this site, Fkh2(S533A)-GFP, and used 2D gels to characterise this protein in stationary phase yeast, growing yeast, and early hyphal cells (Fig. 10A). We found that the Fkh2(S533A)-GFP profile was different from the wild type profile at 40 minutes after hyphal induction, thus supporting the conclusion that Cbk1 targets Fkh2 at this site and that this phosphorylation is necessary for the transit to the early hyphal profile. To provide further evidence that Cbk1 targets Fkh2, and to test whether the action is direct, we carried out an *in vitro* kinase assay. To do this, we used Mob2-HA to immunoprecipitate the Cbk1-Mob2 kinase from an early hyphal lysate and recombinant Fkh2 C-terminal domain fused to GST (GST-Fkh2(CT)) as substrate. Fig. 10B shows that the immune-precipitated Mob2-HA can indeed phosphorylate GST-Fkh2(CT) *in vitro*. Only background levels of signal resulted from negative controls lacking either immuno-precipitated Mob2-HA or the immuno-precipitation products from a mock lysate. Thus, the reaction is specific to Mob2-Cbk1 and is not due to a co-purifying kinase. The *fkh2(S533A)* strain had a normal yeast morphology but showed morphological defects on hyphal induction, thus showing that phosphorylation of Fkh2 S533 by Cbk1-Mob2 is necessary for normal hyphal development (Fig. 10C).

## Discussion

### Fkh2 is phosphorylated in a cell cycle-independent fashion by the action of Cdc28 and Cbk1

The Fork-head transcription factors Fkh1 and Fkh2 have been extensively studied in *S. cerevisiae* where they regulate the G2 transcription program, including the expression of the G2 cyclin gene *CLB*2 required for mitotic entry, and the transcription factor genes *SWI5* and *ACE*2 required for the M to G1 phase gene expression program [53–56]. In *S. cerevisiae*, Fkh2 is phosphorylated by Cdc28-Clb5 during S-phase to activate transcription at the promoters of *CLB*2 cluster genes [43]. Here we show that Fkh2, the single *Sc*Fkh1 and *Sc*Fkh2 homologue in *C. albicans*, undergoes a radical change in its phosphorylation profile within 5 min of hyphal induction. Previously, it has been reported that spindle pole body duplication, which marks the start of the cell cycle, is coincident with the appearance of the septin ring within the germ tube [44]. In the experiments described in Fig. 1, septin rings are only present in hyphae after 60 min, and yet the change in phosphorylation was apparent as early as 5 min, and is disappearing by 60 min. Clearly, Fkh2 phosphorylation triggered by hyphal induction of early G1 cells occurs far before START and is thus cell cycle-independent. In contrast, Fkh2 phosphorylation in yeast cells occurs after bud emergence, consistent with the timing of S-phase phosphorylation reported in *S. cerevisiae* [43].

We initially identified the change in Fkh2 phosphorylation status using 1D gels which suggested that Fkh2 was not phosphorylated in stationary phase but was rapidly phosphorylated upon hyphal induction before the start of the cell cycle. In contrast, 1D gels suggested that Fkh2 in yeast cells was phosphorylated at a later time and only after the start of the cell cycle. However, the increased detail provided by 2D protein gels showed a more complex pattern and revealed that both growing and stationary phase yeast cells contained a complex pool of Fkh2 phospho-isoforms. Importantly, this complex profile underwent a radical, but transient transformation upon hyphal induction.

Mass spectrometry identified that Fkh2 was phosphorylated as sites corresponding to the full Cdc28 consensus target sites. Non-phosphorylatable substitutions at the six full target sites reduced the number of 2D gel peaks in the profile of both yeast and hyphal cells and the shift to the characteristic hyphal profile was no longer evident. However, the transition to the early hyphal profile occurred in Fkh2 carrying phosphomimetic substitutions at these six sites. Thus, phosphorylation of these residues is necessary for the shift to the hyphal profile. Non-phosphorylatable substitutions at all 15 potential full and minimal Cdc28 target sites essentially eliminated all peaks in stationary phase and growing yeast cells, but clear peaks remained in early hyphal cells suggesting the action of a second kinase, the action of which is specific to early hyphal cells. We showed that a strong candidate for this second kinase is Cbk1-Mob2 acting on the S533 residue. An *in vitro* kinase assay and the altered Fkh2 phosphorylation profile in 2D gels when either Cdc28 or Cbk1 activity was inhibited both add support to the conclusion that both Cdc28 and Cbk1 kinases act on Fkh2. We investigated the likely cyclin partner for Cdc28 using mutants in which G1 cyclins were either deleted or depleted. The results suggested that both Cln3 and Ccn1 may be required for the switch to the hyphal-specific pattern. Interestingly, previous reports have shown that cells lacking either Cln3 or Ccn1 initiate hyphal formation but are unable to maintain hyphal growth long term, phenotypes that are reminiscent of the *fkh2(6A)* phenotype which is also unable to maintain the hyphal state [57–59]. In addition, Cdc28-Ccn1 in collaboration with Gin4 phosphorylates the septin Cdc11 on adjacent residues [30]. When this phosphorylation is prevented, hyphal germ tubes form normally but the tip swells and polarized growth ceases after the formation of the first septum.

Taken together these data show that Fkh2 exists as multiple phosphorylated isoforms in both yeast and hyphal growth modes and that Cdc28 is likely to target six key residues and possibly up to a total of 15 residues. However, immediately after hyphal induction there is a transient shift in the phosphorylation profile which is dependent on phosphorylation at the six full Cdc28 target sites. Thus while Fkh2 is phosphorylated in both yeast and early hyphal cells, the phosphorylation state of Fkh2 is qualitatively different at early times after hyphal induction compared to either stationary phase yeast or growing yeast cells. It is important to bear in mind that the complex 2D profiles show that there is a pool of differentially phosphorylated phospho-isoforms of Fkh2 in stationary phase cells, growing yeast and hyphae. With six possible sites that we have shown to be phosphorylated, there are 64 different possible phosphorylated states (2^6^). With 15 possible sites the number of different phosphorylation states is much larger. Our data do not allow us to definitively resolve whether unique sites are phosphorylated in hyphae compared to yeast. However, it is clear that the spectrum of phosphorylated isoforms changes rapidly on hyphal induction in a way that is not observed in yeast, and that this rapid change contributes towards the induction of genes that are required for pathogenesis.

The characteristic early hyphal profile may reflect a change in the spectrum of phosphorylated states of Cdc28 target sites, but the change also depends on the action of Cbk1-Mob2. The change in the phosphorylation pattern of Fkh2(6DE), but not Fkh2(6A), upon hyphal induction suggests that phosphorylation at Cdk1 sites may prime Fkh2 for further phosphorylation by a second kinase. While it is attractive to speculate that Cbk1 phosphorylation is dependent on Cdk1 phosphorylation, we have not addressed this issue experimentally so there is no direct evidence that this is the case. Relatively little is known about the action of Cbk1 and its regulatory subunit, Mob2, despite it being absolutely required for hyphal growth [32]. Moreover, its action is necessary for the expression of hyphal-specific genes [33], consistent with the idea that phosphorylation of Fkh2 by Cbk1 acts to redirect the specificity of Fkh2 to promote hyphal gene expression. Interestingly, Cbk1 has recently been shown to phosphorylate Bcr1, a key transcription factor in biofilm formation [40]. Here we demonstrate another mechanism by which Cbk1 positively regulates biofilm formation, through Fkh2, suggesting that Cbk1 has a general role in promoting biofilm formation.

### Phosphorylation of Fkh2 during hyphal growth redirects Fkh2 specificity

Identification of the sites targeted by Cdc28-Ccn1/Cln3 allowed us to investigate the physiological role of Fkh2 phosphorylation. Transcript profiling showed that the *fkh2*(*6A)* mutant is defective in the expression of genes involved in important aspects of *C. albicans* pathogenicity. In contrast, *fkh2ΔΔ* mutants are defective in a wide range of metabolic functions. Moreover, the gene set whose expression is reduced in *fkh2ΔΔ* shows only a partial overlap with the loss of functions that result from non-phosphorylatable alanine substitutions at the six Cdc28 target sites. Thus, while we confirmed a previous report that Fkh2 plays a role in the expression of hyphal-specific genes [45], we have also revealed that phosphorylation of Fkh2 is specifically required for the expression of genes that promote pathogenesis within the host. These include: *SAP4,6* which are expressed during mucosal and systemic infections for nitrogen utilisation from host proteins; *HYR1* which encodes a GPI anchored cell wall protein required for protection against killing of *C. albicans* by neutrophils [60]; *HGC1*, which is required for hyphal growth, biofilm formation, and virulence in a mouse model of systemic infection [25]; and *SUN41* which is required for biofilm formation [47]. Expression of these genes has previously been shown to be regulated through the cAMP-PKA and MAPK pathways. Thus we have identified a new pathway, through Cdc28-Ccn1/Cln3 and Cbk1 that acts to positively regulate hyphal gene expression alongside the aforementioned pathways. Importantly, we have also shown that the change in gene expression observed in the *fkh2(6A)* mutant was associated with a corresponding reduction of *C. albicans* virulence functions such as: hyphal maintenance, host cell damage, biofilm formation and host immune response activation. Our work suggests that Cdc28-Ccn1/Cln3 positively regulates *HGC1* expression on hyphal induction via Fkh2. This early activation of *HGC1* expression through Fkh2 may be a key role of Ccn1 and Cln3 on hyphal induction, and thus could explain why Fkh2 is only phosphorylated for a short period on hyphal induction.

### Phosphorylation of Fkh2 controls the interaction with the chromatin modifier Pob3

Here we have identified that Fkh2 physically associates with Pob3 in a manner that is dependent on Fkh2 phosphorylation. Pob3 in *S. cerevisiae* is a subunit of the heterodimeric FACT complex that reorganizes nucleosomes to allow access of RNA polymerase II and DNA polymerase to promoters, thus promoting transcription initiation and DNA replication [51]. Interestingly, a role for *Sc*Fkh2 in controlling origin firing has recently been reported [61]. The *fkh2(6A)* mutant initiates germ tube formation normally, but fails to maintain hyphal growth and is defective in agar invasion. Chromatin remodelling has been shown to play a key role in hyphal maintenance [62, 63]. Significantly, we observe a peak in Fkh2 phosphorylation after 40 min of hyphal induction, which is consistent with the timing of chromatin reorganisation that results in the ejection of the repressor Nrg1 from the promoter of hyphal-expressed genes, a requirement for the long-term maintenance of hyphal growth [62, 63]. This suggests that phosphorylation of Fkh2 on hyphal induction may be required to direct Pob3 to the promoters of hyphal specific genes, in order to regulate the remodelling events that are required to sustain hyphal and pathogenic gene expression.

### Conclusion

In this study, we identified how the *C. albicans* Fkh2 transcription factor has evolved to acquire a new function in addition to its conserved housekeeping role. *Ca*Fkh2 switches from cell cycle-dependent phospho-regulation during yeast growth, to a specific early phospho-regulatory event during hyphal growth that is independent of the cell cycle. This early hyphal phosphorylation is necessary for the positive regulation of genes involved in invasive growth and pathogenesis. Therefore, we can propose a new mechanism, by which *C. albicans* specifically modifies a key component of its cell cycle transcription machinery by phosphorylation, in the switch from commensalism to pathogenicity.

## Materials and Methods

### Media and growth conditions

*C. albicans* cells were routinely grown in 1% yeast extract, 2% peptone and either 2% glucose (YEPD) or galactose (YEPG), or in 0.67% yeast nitrogen base without amino acids and 2% glucose (GMM) with appropriate amino acids for auxotrophic mutants. For yeast growth, cells were diluted to OD_600_ = 0.6 in pH 4.0 YEPD media and incubated at 30°C. For hyphal induction, cells were washed in sterile water and diluted to OD_600_ = 0.6 in pH 7.0 YEPD media supplemented with 10–20% fetal calf serum (FCS) and incubated at 37°C. G1 cells were obtained by centrifugal elutriation from a log phase YEPD culture using a Beckman elutriating rotor JE 5.0. For *MET3* promoter shutdown, methionine and cysteine were added to a final concentration of 2.5 mM and 0.5 mM respectively. Inhibition of the analogue sensitive *cdc28-as1* allele was carried out by adding 1NM-PP1 (Merck-Calbiochem) to a final concentration of 30 μM.

### Strain and plasmid construction

*C. albicans* strains, plasmids and primers are listed in the S1 Table. Strains were generated either by PCR-generated fragment recombination or plasmid integration as previously described [64–66]. Site-directed mutagenesis was carried out using the QuickChange Multi Site-Directed mutagenesis kit (Agilent technologies, Edinburgh, UK) following the manufacturer’s instructions.

### Protein extraction, phosphatase treatment and western blotting

Cells were harvested by centrifugation, washed once with sterile water and then re-suspended in two volumes of lysis buffer (50 mM Tris-HCl pH 7.5. 100 mM NaCl, 0.1% Triton X-100 and 0.1% w/v sodium deoxycholate) containing a protease inhibitor cocktail (Roche) and phosphatase inhibitors (50 mM NaF and 100 mM β-glycerophosphate). One cell volume of 0.4 mm glass beads were then added before 3 rounds of 30 second homogenization in a mini-bead beater (Biospec products, Bartlesville, OK,USA), with 2 min on ice in between. Lysates were then cleared by centrifugation at 13,000 rpm for 10 min. For direct Western Blotting, 30–50 μg protein aliquots were resolved by 1D PAGE. For Immuno-precipitation (IP) 2 mg of total protein was incubated with 50 μl of protein-G Dynabeads (Life Technologies, Carlsbad CA USA), after pre-binding with GFP (Roche) or Myc/HA (Bioserv, Sheffield UK) mouse monoclonal antibodies, in a total IP volume of 500 μl for 90 min at 4°C. IP products were washed 2–4 times with 10 bead volumes of cold lysis buffer before being re-suspended and boiled for 5 min in 48 μl of 1x protein loading dye. αPSTAIR loading control is a monoclonal antibody raised against a synthetic peptide (P7962 Sigma). αPser CDK (Cell-Signalling 2324S) recognises phosphorylated serines in the Cdc28 consensus target motif SPxR/K.

Dephosphorylation of proteins was carried out using Lambda Phosphatase (NEB # P0753). 400 units of lambda phosphatase were used for 40 μl of 10 mg/ml lysate in a total reaction volume of 50 μl, including 1mM MnCl_2_ and 1x PMP buffer. The reaction was carried out at 30°C for 1 h on a shaking platform set at 200 rpm

Proteins were visualised on Western blots using ECL (GE Healthcare, Amersham, UK) and resulting chemifluorescence recorded using a GeneGnome (Syngene, Cambridge, UK)

### *In vitro* kinase assay

C-terminal fragments of the Fkh2 wild-type sequence or substituted with alanine at the Cdc28 target site were expressed and purified from *E. coli* as previously described [28]. Cdc28 or Cbk1 was purified from 2 mg lysate in a strain where both alleles were fused to HA and then washed three times with lysis buffer containing 750 mM NaCl and once with standard lysis buffer. 25 μl of 2x kinase assay buffer (100 mM Tris-HCl pH 7.5, 2 mM EGTA, 0.02% v/v Tween-20 (Sigma-Aldrich) 2 mM DTT, 2 mM β-glycerophosphate and 20 mM MgCl_2_) was added to the beads along with 24 μl of 1 mg/ml GST fragment and 1 μl ATP from a 5 mM stock. Reactions were incubated at 37°C for 1 h with gentle agitation. The reaction was quenched by boiling in 1x loading dye for 5 min.15 μl of reaction was separated by 10% 1D PAGE before subsequent Western blotting to PVDF membrane. Kinase assay blots were initially probed with either αP_SER_(CDK) (Cell-Signalling 2324S) or αP_SER_ Q5 (Qiagen 37430), and then stripped and re-probed with rabbit monoclonal αGST (Santa-Cruz Biotechnology)

### Mass spectrometry

Fkh2-HA or Fkh2-Myc was immuno-purified from approximately 1 g of total cell lysate using 60 μl of the appropriate Ez-view agarose slurry (Sigma-Aldrich). The protein-bound agarose was washed four times with cold lysis buffer before boiling for 10 min in 30 μl 1x protein loading dye. The denatured proteins were separated on a 4–20% gradient SDS-PAGE gel (Bio-Rad) and then stained using Instant Blue™ Coomassie stain (Expedeon). Bands of interest were excised for subsequent MS analysis as previously described [28].

### 2-Dimensional (2D) Gel Electrophoresis

2D electrophoresis was carried out as previously described [67] with modifications. Fkh2-GFP was purified from 2 mg of total protein by IP, and then removed from the beads by adding 100 μl of hydration buffer with 4 mM DTT and incubating for 1 h at room temperature before subsequent isoelectric focusing (IEF) using the IPGphor3 IEF system (GE Life-sciences) on a pH 3–10 range IPG strip. Western blots were imaged with a GeneGnome (Syngene, Cambridge UK). The images shown in Figs. 1E, 2E and 3B and 10A are 400×70 pixels aligned 100 pixels from the fixed marker in a 1.3 MP image. The intensities of the fluorescence in these images were profiled using the FIJI distribution of ImageJ (http://fiji.sc/Fiji). The profiles were derived from a line that was manually drawn through the signal and the resulting intensity values exported as csv files into Microsoft Excel (Microsoft Corporation, US) to generate the intensity plots shown above the autoradiograms of the 2D gels.

### RNA isolation, microarray and analysis

Log phase yeast and 3 h post-induction hyphal cells were harvested and the pellets were snap-frozen in liquid nitrogen. RNA was extracted using the RNeasy minikit (QIAGEN) following the manufacturer’s instructions for yeast mechanical disruption, using a TOMY Microsmash (TOMY Digital Biology, Tokyo JP). Total RNA was treated with DNase I (Roche) before RNA-Cleanup using the RNeasy kit. 10 µg of total RNA was reverse-transcribed with either Cy3 (control) or Cy5 (experimental) dyes and hybridised to full *C. albicans* arrays from Microarrays Inc. (Huntsville Alabama). Arrays were subsequently scanned with an Axon GenePix 4000B scanner. Duplicates of each array were carried out, with each array containing multiple replicate probes.

Microarray data was analysed using the limma (version 3.21.18) package for the R language (version 3.1.1) [68]. Briefly, after quality control the arrays were background normalised using the “normexp” method [69] and spots with an intensity of less than 50 over background in both channels were removed. Arrays were then normalized using the “print-tip loess” method [70]. Within array replicate spots were averaged. Differentially expressed genes between wild type, *fkh2(6A)* and *fkh2ΔΔ* yeast samples were assessed using empirical Bayes moderated t-tests [71]. For hyphal samples we fitted a linear model with indicator variables for genotype and used moderated t-statistics to test the significance of the coefficients for the phosphorylation mutant and deletion genotype, as well as the significance of the difference between these coefficients. False discovery rate was assessed across all tests simultaneously using the method of Benjamini and Hochberg [72]. Where multiple probes targeted the same gene, the least significant was selected. Genes were regarded as more down regulated in *fkh2(6A)* than *fkh2ΔΔ* if: a) The gene was down-regulated more than two fold at the 5% FDR threshold in the *fkh2(6A)* samples compared to wild type b) the fold change was less negative (or was positive) in the *fkh2ΔΔ* samples and c) the difference in fold changes was significant at the 5% FDR threshold. Similar criteria were used for genes more down regulated in *fkh2ΔΔ* samples. Data and detail analysis protocol are deposited in GEO with accession GSE64383. GO analysis was conducted using the GO Stats R/Bioconductor package [73], using conditional testing (account for GO graph structure) and calculating FDR using the Benjamini and Hochberg method [72].

### qPCR

Total RNA was prepared as above, and 0.5 μg was reverse-transcribed using Superscript III (Invitrogen) in a reaction volume of 30 μl following the manufacturer’s instructions. The mock reverse transcription reaction contained everything except the Superscript enzyme. Reaction mixes were then diluted two-fold for use in subsequent qPCR reactions. Triplicate qPCR reactions were carried out using 0.5 μM of the primer pairs listed in the supplementary materials in a total 10 μl volume with 2x Sensi-mix (Bioline). Samples were run on the Rotor-gene-6000 system (QIAGEN). Normalisation of expression levels was carried out using the *ADE2* gene, before comparative ΔΔCT analysis of expression levels in wild-type and mutant strains.

### Microscopy

Fluorescence microscopy was carried out as previously stated [67]

### Biofilm formation

These assays were carried out as previously described [74]

### Epithelial cell damage and immune activation assays

**Cell culture**. TR146 oral (buccal) epithelial cells [75] (SkinEthic Laboratories, Lyon FR) were maintained in DMEM (Sigma) supplemented with 10% (v/v) heat-inactivated foetal bovine serum (Sigma), 1% (v/v) penicillin-streptomycin solution and cultured at 37°C, 5% CO_2_. All experiments were performed in serum-free DMEM (Sigma). *C. albicans* strains were cultured overnight in YEPD medium at 30°C, 200 rpm. Cells were collected by centrifugation and washed twice in sterile PBS prior to use in epithelial cell damage assays.

### Epithelial cell damage assay

Confluent TR146 monolayers were infected with *C. albicans* strains (multiplicity of infection = 0.01) and cultured for 24 h (37°C, 5% CO_2_). Cell culture supernatants were collected and epithelial cell damage was determined by quantification of lactate dehydrogenase (LDH) activity using the Cytox 96 Non-Radioactive Cytotoxicity Assay kit (Promega) according to the manufacturer’s instructions. Recombinant porcine LDH (Sigma) was used to generate a standard curve.

### Cytokine determination

TR146 monolayers were infected with *C. albicans* strains as described above. Cell culture supernatants were collected and cytokines quantified using the Fluorokine MAP cytokine multiplex kit (R&D Systems), coupled with a Bio-Plex™ 200 machine according to the manufacturer’s instructions. The trimmed median value was used to derive standard curves and calculate sample concentrations

## Supporting Information

S1 Fig Initial screen of CDK target proteins. Yeast and hyphal time-courses were carried out for Orf19.3469-GFP (A) Orf19.1948-GFP (B) and Fkh2-YFP (C). 30 μg total protein was run for each time point. In A and B αPSTAIRE was used as a control for equal loading.
(TIF)

S2 Fig Demonstration of reproducibility of 2D gels. Independent replication of the experiment shown in Fig. 1. Replicate results are shown on the profiles as a solid black line, with the grey dashed line representing the initial profile from Fig. 1. The top panels of gels are those from Fig. 1, with the replicates present in the bottom panel.
(TIF)

S3 Fig Mass-spectrometry phospho-peptide mapping data. A) Coomassie Blue stained gel of Fkh2-HA purified from 5 L of culture 40 min after hyphae were induced from unbudded stationary phase yeast cells. Bands A1 and A2 indicate those excised for MS analysis. B) List of phospho-peptides detected with phosphorylation occurring in Cdc28 consensus motifs. C) All phospho-sites detected by phospho-peptide mapping. Red—significant hit, Grey—possible phosphorylation site, Green—either site is phosphorylated. D) Mutation of significant hits from phospho-peptide mapping does not affect Fkh2’s associated phospho-shift. *FKH2-YFP* and *fkh2(6AMS)-GFP* strains were grown for 80 min in yeast or 40 min hyphal growth conditions before harvesting cells for protein extraction. E) Yeast and hyphal 1D phosphorylation profiles of Cdc28 consensus target site mutants with phosphoacceptor residues mutated to alanine or glutamate/aspartate. F) Mutation of all CDK consensus and minimal sites (15A and 15DE), or all Cdc28 target sites C-terminal to the DNA binding domain in Fkh2 (10A). Strains were grown and samples processed as previously described.
(TIF)

S4 Fig Fkh2 over-expression leads to nuclear defects. *FKH2-YFP* and *P_GAL1_-FKH2-GFP* were grown to stationary phase in glucose rich media. The strains were then re-inoculated into yeast growth conditions in either glucose (YEPD) or galactose (YEPG) rich media and grown for 3 h at 30°C. A: Cells were washed in PBS, DAPI was added and then GFP/DAPI fluorescence images were taken at x100 magnification. Scale bars represent 10 μm. B: Western blot using a monoclonal antibody to GFP shows that *FKH2-GFP* was overexpressed from the *GAL1* promoter compared to the control *FKH2-YFP* expressed from its native promoter.
(TIF)

S5 Fig The search for other kinases that act on Fkh2. Fkh2 was C-terminally tagged with GFP in a series of kinase mutants generously provided by their original constructors: Tpk1 [77]; Ssn3 [78]; Pho85-sd [79]; Hog1 [80]; Cek1 [81]; Yak1 [82]. Stationary phase yeast cells were induced to form hyphae as described in materials and methods. Panels A,B cells were isolated 40 minutes after hyphal induction and the phosphorylation state compared to the wild type by the presence of a band shift in a Western blot using a monoclonal antibody against GFP. C-G) Western blots were prepared from stationary phase samples (0) and samples prepared 40 minutes after hyphal induction (H40). C) Pho85 expressed from the Tet-off promoter was repressed by the addition of 20µg.ml^-1^ Doxycyclin. G) Only the *cbk1Δ* strain failed to show the double band 40 minutes after hyphal induction, being present as the stationary phase or phosphatase treated form. All the other strains showed the same double band as the wild type.
(TIF)

S1 Dataset Fkh2 phospho-peptides. Excel spreadsheet containing the full list of phospho-peptides of Fkh2 from the experiment in Fig. 2A.
(XLSX)

S2 Dataset Combined microarray data for all Fkh2 mutants. Excel spreadsheet containing combined microarray profiles for the effect of *fkh2ΔΔ* and *fkh2(6A)* on both yeast and hyphal growth, and *FKH2* over-expression under yeast growth conditions.
(XLSX)

S1 Table Strains, Plasmids and primers used. (DOCX)

## References

[1] Perlroth J, Choi B, Spellberg B (2007) Nosocomial fungal infections: epidemiology, diagnosis, and treatment. Medical Mycology 45: 321–346. 10.1080/13693780701218689 17510856

[2] Kullberg BJ, Filler SG (2002) Candidemia. In: Calderone RA, editors. *Candida* and Candidiasis. Washington DC: ASM Press pp. 327–340.

[3] Runke M (2002) Skin and mucous infections. In: Calderone R, editors. Candida and Candidiasis. Washington: ASM Press pp. 307–325.

[4] Sudbery PE, Gow NAR, Berman J (2004) The distinct morphogenic states of *Candida albicans* . Trends Microbiol 12: 317–324. 10.1016/j.tim.2004.05.008 15223059

[5] Sudbery PE (2011) Growth of *Candida albicans* hyphae. Nat Rev Microbiol 9: 737–748. 10.1038/nrmicro2636 21844880

[6] Gow NA, van de Veerdonk FL, Brown AJ, Netea MG (2012) Candida albicans morphogenesis and host defence: discriminating invasion from colonization. Nat Rev Microbiol 10: 112–122. 10.1038/nrmicro2711 PMC3624162 22158429

[7] Moyes DL, Runglall M, Murciano C, Shen C, Nayar D, Thavaraj S, Kohli A, Islam A, Mora-Montes H, Challacombe SJ, Naglik JR (2010) A Biphasic Innate Immune MAPK Response Discriminates between the Yeast and Hyphal Forms of Candida albicans in Epithelial Cells. Cell Host & Microbe 8: 225–235. 10.1016/j.chom.2010.08.002 20833374 PMC2991069

[8] Ray TL, Payne CD (1988) Scanning electron-microscopy of epidermal adherence and cavitation in murine candidiasis—a role for *Candida* acid proteinase. Infect Immun 56: 1942–1949. 3294180 10.1128/iai.56.8.1942-1949.1988 PMC259505

[9] Scherwitz C (1982) Ultrastructure of Human Cutaneous Candidosis. Journal of Investigative Dermatology 78: 200–205. 10.1111/1523-1747.ep12506451 7035576

[10] Dalle F, Wächtler B, L’Ollivier C, Holland G, Bannert N, Wilson D, Labruère C, Bonnin A, Hube B (2010) Cellular interactions of *Candida albicans* with human oral epithelial cells and enterocytes. Cellular Microbiology 12: 248–271. 10.1111/j.1462-5822.2009.01394.x 19863559

[11] Park H, Myers CL, Sheppard DC, Phan QT, Sanchez AA, Edwards JE, Filler SG (2005) Role of the fungal Ras-protein kinase A pathway in governing epithelial cell interactions during oropharyngeal candidiasis. Cellular Microbiology 7: 499–510. 10.1111/j.1462-5822.2004.00476.x 15760450

[12] Phan QT, Myers CL, Fu Y, Sheppard DC, Yeaman MR, Welch WH, Ibrahim AS, Edwards JE Jr., Filler SG (2007) Als3 Is a *Candida albicans* Invasin That Binds to Cadherins and Induces Endocytosis by Host Cells. PLoS Biol 5: e64 10.1371/journal.pbio.0050064 17311474 PMC1802757

[13] Zakikhany K, Naglik JR, Schmidt-Westhausen A, Holland G, Schaller M, Hube B (2007) In vivo transcript profiling of Candida albicans identifies a gene essential for interepithelial dissemination. Cellular Microbiology 9: 2938–2954. 10.1111/j.1462-5822.2007.01009.x 17645752

[14] Lorenz MC, Bender JA, Fink GR (2004) Transcriptional Response of *Candida albicans* upon Internalization by Macrophages. Euk Cell 3: 1076–1087. 10.1128/EC.3.5.1076-1087.2004 15470236 PMC522606

[15] Grubb SEW, Murdoch C, Sudbery PE, Saville SP, Lopez-Ribot JL, Thornhill MH (2009) Adhesion of *Candida albicans* to Endothelial Cells under Physiological Conditions of Flow. Infect Immun 77: 3872–3878. 10.1128/IAI.00518-09 19581400 PMC2738003

[16] Finkel JS, Mitchell AP (2011) Genetic control of *Candida albicans* biofilm development. Nat Rev Microbiol 9: 109–118. 10.1038/nrmicro2475 21189476 PMC3891587

[17] Wächtler B, Wilson D, Haedicke K, Dalle F, Hube B (2011) From Attachment to Damage: Defined Genes of *Candida albicans* Mediate Adhesion, Invasion and Damage during Interaction with Oral Epithelial Cells. PLoS ONE 6: e17046 10.1371/journal.pone.0017046 21407800 PMC3044159

[18] Sanglard D, Hube B, Monod M, Odds FC, Gow NAR (1997) A triple deletion of the secreted aspartyl proteinase genes SAP4, SAP5, and SAP6 of Candida albicans causes attenuated virulence. Infect Immun 65: 3539–3546. 9284117 10.1128/iai.65.9.3539-3546.1997 PMC175504

[19] Hoyer LL, Payne TL, Bell M, Myers AM, Scherer S (1998) *Candida albicans* ALS3 and insights into the nature of the *ALS* gene family. Curr Genet 33: 451–459. 10.1007/s002940050359 9644209

[20] Staab JF, Bradway SD, Fidel PL, Sundstrom P (1999) Adhesive and mammalian transglutaminase substrate properties of *Candida albicans* Hwp1. Science 283: 1535–1538. 10.1126/science.283.5407.1535 10066176

[21] Carlisle PL, Kadosh D (2013) A genome-wide transcriptional analysis of morphology determination in *Candida albicans* . Mol Biol Cell 24: 246–260. 10.1091/mbc.E12-01-0065 23242994 PMC3564527

[22] Kadosh D, Johnson AD (2005) Induction of the *Candida albicans* filamentous growth program by relief of transcriptional repression: a genome-wide analysis. Mol Biol Cell 16: 2903–2912. 10.1091/mbc.E05-01-0073 15814840 PMC1142434

[23] Nantel A, Dignard D, Bachewich C, Harcus D, Marcil A, Bouin AP, Sensen CW, Hogues H, het Hoog M, Gordon P, Rigby T, Benoit F, Tessier DC, Thomas DY, Whiteway M (2002) Transcription profiling of *Candida albicans* cells undergoing the yeast-to-hyphal transition. Mol Biol Cell 13: 3452–3465. 10.1091/mbc.E02-05-0272 12388749 PMC129958

[24] Shapiro RS, Robbins N, Cowen LE (2011) Regulatory Circuitry Governing Fungal Development, Drug Resistance, and Disease. Microbiol Mol Biol Rev 75: 213–267. 10.1128/MMBR.00045-10 21646428 PMC3122626

[25] Zheng X, Wang Y, Wang Y (2004) Hgc1, a novel hypha-specific G1 cyclin-related protein regulates *Candida albicans* hyphal morphogenesis. EMBO J 23: 1845–1856. 10.1038/sj.emboj.7600195 15071502 PMC394249

[26] Court H, Sudbery P (2007) Regulation of Cdc42 GTPase activity in the formation of hyphae in *Candida albicans* . Mol Biol Cell 18: 265–281. 10.1091/mbc.E06-05-0411 17093060 PMC1751335

[27] Zheng XD, Lee RTH, Wang YM, Lin QS, Wang Y (2007) Phosphorylation of Rga2, a Cdc42 GAP, by CDK/Hgc1 is crucial for *Candida albicans* hyphal growth. EMBO J 26: 3760–3769. 10.1038/sj.emboj.7601814 17673907 PMC1952229

[28] Zeng G, Wang YM, Wang Y (2012) Cdc28−Cln3 phosphorylation of Sla1 regulates actin patch dynamics in different modes of fungal growth. Mol Biol Cell. 10.1091/mbc.E12-03-0231 22787279 PMC3431942

[29] Bishop A, Lane R, Beniston R, Lazo B, Smythe C, Sudbery P (2010) Hyphal growth in *Candida albicans* requires the phosphorylation of Sec2 by the Cdc28-Ccn1/Hgc1 kinase. EMBO J 29: 2930–2942. 10.1038/emboj.2010.158 20639857 PMC2944046

[30] Sinha I, Wang YM, Philp R, Li CR, Yap WH, Wang Y (2007) Cyclin-dependent kinases control septin phosphorylation in *Candida albicans* hyphal development. Developmental Cell 13: 421–432. 10.1016/j.devcel.2007.06.011 17765684

[31] Tamaskovic R, Bichsel SJ, Hemmings BA (2003) NDR family of AGC kinases—essential regulators of the cell cycle and morphogenesis. FEBS Letters 546: 73–80. 10.1016/S0014-5793(03)00474-5 12829239

[32] McNemar MD, Fonzi WA (2002) Conserved serine/threonine kinase encoded by *CBK*1 regulates expression of several hypha-associated transcripts and genes encoding cell wall proteins in *Candida albicans* . J Bacteriol 184: 2058–2061. 10.1128/JB.184.7.2058-2061.2002 11889116 PMC134915

[33] Song Y, Cheon SA, Lee KE, Lee SY, Lee BK, Oh DB, Kang HA, Kim JY (2008) Role of the RAM Network in Cell Polarity and Hyphal Morphogenesis in Candida albicans. Mol Biol Cell 19: 5456–5477. 10.1091/mbc.E08-03-0272 18843050 PMC2592677

[34] Maerz S, Seiler S (2010) Tales of RAM and MOR: NDR kinase signaling in fungal morphogenesis. Curr Opin Microbiol 13: 663–671. 10.1016/j.mib.2010.08.010 20869909

[35] Hou MC, Wiley DJ, Verde F, McCollum D (2003) Mob2p interacts with the protein kinase Orb6p to promote coordination of cell polarity with cell cycle progression. J Cell Sci 116: 125–135. 10.1242/jcs.00206 12456722

[36] Yarden O, Plamann M, Ebbole DJ, Yanofsky C (1992) Cot-1, A Gene Required for Hyphal Elongation in Neurospora-Crassa, Encodes A Protein-Kinase. Embo Journal 11: 2159–2166. 1534751 10.1002/j.1460-2075.1992.tb05275.x PMC556683

[37] Weiss EL, Kurischko C, Zhang C, Shokat K, Drubin DG, Luca FC (2002) The *Saccharomyces cerevisiae* Mob2p-Cbk1p kinase complex promotes polarized growth and acts with the mitotic exit network to facilitate daughter cell-specific localization of Ace2p transcription factor. J Cell Biol 158: 885–900. 10.1083/jcb.200203094 12196508 PMC2173146

[38] Bidlingmaier S, Weiss EL, Seidel C, Drubin DG, Snyder M (2001) The Cbk1p pathway is important for polarized cell growth and cell separation in Saccharomyces cerevisiae. Mol Cell Biol 21: 2449–2462. 10.1128/MCB.21.7.2449-2462.2001 11259593 PMC86877

[39] Nelson B, Kurischko C, Horecka J, Mody M, Nair P, Pratt L, Zougman A, Mcbroom LDB, Hughes TR, Boone C, Luca FC (2003) RAM: A conserved signaling network that regulates Ace2p transcriptional activity and polarized morphogenesis. Mol Biol Cell 14: 3782–3803. 10.1091/mbc.E03-01-0018 12972564 PMC196567

[40] Gutierrez-Escribano P, Zeidler U, Belen SM, Bachellier-Bassi S, Clemente-Blanco A, Bonhomme J, Vazquez de Aldana CR, d’Enfert C, Correa-Bordes J (2012) The NDR/LATS Kinase Cbk1 Controls the Activity of the Transcriptional Regulator Bcr1 during Biofilm Formation in Candida albicans. Plos Pathogens 8 10.1371/journal.ppat.1002683 22589718 PMC3349750

[41] Zhu GF, Spellman PT, Volpe T, Brown PO, Botstein D, Davis TN, Futcher B (2000) Two yeast forkhead genes regulate the cell cycle and pseudohyphal growth. Nature 406: 90–94. 10.1038/35021046 10894548

[42] Costanzo M, Schub O, Andrews B (2003) G(1) transcription factors are differentially regulated in Saccharomyces cerevisiae by the Swi6-binding protein Stb1. Mol Cell Biol 23: 5064–5077. 10.1128/MCB.23.14.5064-5077.2003 12832490 PMC162210

[43] Pic-Taylor A, Darieva Z, Morgan BA, Sharrocks AD (2004) Regulation of cell cycle-specific gene expression through cyclin-dependent kinase-mediated phosphorylation of the forkhead transcription factor Fkh2p. Mol Cell Biol 24: 10036–10046. 10.1128/MCB.24.22.10036-10046.2004 15509804 PMC525469

[44] Finley KR, Berman J (2005) Microtubules in *Candida albicans* hyphae drive nuclear dynamics and connect cell cycle progression to morphogenesis. Euk Cell 4: 1697–1711. 10.1128/EC.4.10.1697-1711.2005 16215177 PMC1265902

[45] Bensen ES, Filler SG, Berman J (2002) A forkhead transcription factor is important for true hyphal as well as yeast morphogenesis in *Candida albicans* . Euk Cell 1: 787–798. 10.1128/EC.1.5.787-798.2002 12455696 PMC126749

[46] Chauvel M, Nesseir A, Cabral V, Znaidi S, Goyard S, Bachellier-Bassi S, Firon A, Legrand M, Diogo D, Naulleau C, Rossignol T, d’Enfert C (2012) A Versatile Overexpression Strategy in the Pathogenic Yeast *Candida albicans*: Identification of Regulators of Morphogenesis and Fitness. PLoS ONE 7: e45912 10.1371/journal.pone.0045912 23049891 PMC3457969

[47] Hiller E, Heine S, Brunner H, Rupp S (2007) *Candida albicans* Sun41p, a putative glycosidase, is involved in morphogenesis, cell wall biogenesis, and biofilm formation. Euk Cell 6: 2056–2065. 10.1128/EC.00285-07 17905924 PMC2168408

[48] Leng P, Sudbery PE, Brown AJP (2000) Rad6p represses yeast-hypha morphogenesis in the human fungal pathogen Candida albicans. Mol Microbiol 35: 1264–1275. 10.1046/j.1365-2958.2000.01801.x 10712706

[49] Gow NAR, Gooday GW (1984) A Model for the Germ Tube Formation and Mycelial Growth Form of *Candida albicans* . Sabouraudia-Journal of Medical and Veterinary Mycology 22: 137–143. 10.1080/00362178485380211 6374934

[50] Tabb MM, Tongaonkar P, Vu L, Nomura M (2000) Evidence for separable functions of Srp1p, the yeast homolog of importin alpha (Karyopherin alpha): Role for Srp1p and Sts1p in protein degradation. Mol Cell Biol 20: 6062–6073. 10.1128/MCB.20.16.6062-6073.2000 10913188 PMC86082

[51] Formosa T (2008) FACT and the reorganized nucleosome. Molecular Biosystems 4: 1085–1093. 10.1039/b812136b 18931784

[52] Mazanka E, Alexander J, Yeh BJ, Charoenpong P, Lowery DM, Yaffe M, Weiss EL (2008) The NDR/LATS family kinase Cbk1 directly controls transcriptional asymmetry. Plos Biology 6: 1778–1790. 10.1371/journal.pbio.0060203 18715118 PMC2517623

[53] Hollenhorst PC, Bose ME, Mielke MR, Muller U, Fox CA (2000) Forkhead genes in transcriptional silencing, cell morphology and the cell cycle: Overlapping and distinct functions for FKH1 and FKH2 in Saccharomyces cerevisiae. Genetics 154: 1533–1548. 10747051 10.1093/genetics/154.4.1533 PMC1461039

[54] Hollenhorst PC, Pietz G, Fox CA (2001) Mechanisms controlling differential promoter-occupancy by the yeast forkhead proteins Fkh1p and Fkh2p: implications for regulating the cell cycle and differentiation. Gene Dev 15: 2445–2456. 10.1101/gad.906201 11562353 PMC312786

[55] Hollenhorst PC, Bose ME, Mielke MR, Muller U, Fox CA (2000) Forkhead genes in transcriptional silencing, cell morphology and the cell cycle: Overlapping and distinct functions for FKH1 and FKH2 in Saccharomyces cerevisiae. Genetics 154: 1533–1548. 10747051 10.1093/genetics/154.4.1533 PMC1461039

[56] Zhu GF, Spellman PT, Volpe T, Brown PO, Botstein D, Davis TN, Futcher B (2000) Two yeast forkhead genes regulate the cell cycle and pseudohyphal growth. Nature 406: 90–94. 10.1038/35021046 10894548

[57] Bachewich C, Nantel A, Whiteway M (2005) Cell cycle arrest during S or M phase generates polarized growth via distinct signals in *Candida albicans* . Mol Microbiol 57: 942–959. 10.1111/j.1365-2958.2005.04727.x 16091036

[58] Chapa Y, Lazo B, Lee S, Regan H, Sudbery P (2011) The mating projections of Saccharomyces cerevisiae and Candida albicans show key characteristics of hyphal growth. Fungal Biology 115: 547–556. 10.1016/j.funbio.2011.02.001 21640318

[59] Loeb JJ, Sepulveda-Becerra M, Hazan I, Liu HP (1999) A G1 cyclin is necessary for maintenance of filamentous growth in *Candida albicans* . Mol Cell Biol 19: 4019–4027. 10330142 10.1128/mcb.19.6.4019 PMC104361

[60] Luo G, Ibrahim AS, Spellberg B, Nobile CJ, Mitchell AP, Fu Y (2010) Candida albicans Hyr1p Confers Resistance to Neutrophil Killing and Is a Potential Vaccine Target. Journal of Infectious Diseases 201: 1718–1728. 10.1086/652407 20415594 PMC3933264

[61] Knott SR, V, Peace JM, Ostrow A, Gan Y, Rex AE, Viggiani CJ, Tavare S, Aparicio OM (2012) Forkhead Transcription Factors Establish Origin Timing and Long-Range Clustering in S. cerevisiae. Cell 148: 99–111. 10.1016/j.cell.2011.12.012 22265405 PMC3266545

[62] Lu Y, Su C, Liu H-P (2012) A GATA Transcription Factor Recruits Hda1 in Response to Reduced Tor1 Signaling to Establish a Hyphal Chromatin State in Candida albicans. Plos Pathogens 8 10.1371/journal.ppat.1002663 22536157 PMC3334898

[63] Lu Y, Su C, Wang A, Liu H-P (2011) Hyphal Development in *Candida albicans* Requires Two Temporally Linked Changes in Promoter Chromatin for Initiation and Maintenance. Plos Biology 9 10.1371/journal.pbio.1001105 21811397 PMC3139633

[64] Gola S, Martin R, Walther A, Dunkler A, Wendland J (2003) New modules for PCR-based gene targeting in *Candida albicans*: rapid and efficient gene targeting using 100 bp of flanking homology region. Yeast 20: 1339–1347. 10.1002/yea.1044 14663826

[65] Li CR, Lee RT-H, Wang YM, Zheng XD, Wang Y (2007) Candida albicans hyphal morphogenesis occurs in Sec3p-independent and Sec3p-dependent phases separated by septin ring formation. J Cell Sci 120: 1898–1907. 10.1242/jcs.002931 17504812

[66] Schaub Y, Dunkler A, Walther A, Wendland J (2006) New pFA-cassettes for PCR-based gene manipulation in *Candida albicans* . Journal of Basic Microbiology 46: 416–429. 10.1002/jobm.200510133 17009297

[67] Caballero-Lima D, Sudbery PE (2014) In *Candida albicans*, phosphorylation of Exo84 by Cdk1-Hgc1 is necessary for efficient hyphal extension. Mol Biol Cell 25: 1097–1110. 10.1091/mbc.E13-11-0688 24501427 PMC3967973

[68] Smyth GK (2005) Limma: Linear models for microarray data. In: Gentalman R, Carey V, Huber W, editors. Bioinformatics and Computational Biology Solution Using R and Bioconductor. pp. 397–420.

[69] Ritchie ME, Silver J, Oshlack A, Holmes M, Diyagama D, Holloway A, Smyth GK (2007) A comparison of background correction methods for two-colour microarrays. Bioinformatics 23: 2700–2707. 10.1093/bioinformatics/btm412 17720982

[70] Smyth GK, Speed T (2003) Normalization of cDNA microarray data. Methods 31: 265–273. 10.1016/S1046-2023(03)00155-5 14597310

[71] Smyth GK (2005) Linear models and empirical Bayes methods for assessing differential expression in microarray experiments. Statistical Applications in Genetics and Molecular Biology 3: Article 3 10.2202/1544-6115.1027 16646809

[72] Benjamini Y, Hochberg Y (1995) Controlling the False Discovery Rate—A Practical and Powerful Approach to Multiple Testing. Journal of the Royal Statistical Society Series B-Methodological 57: 289–300.

[73] Falcon S, Gentleman R (2007) Using GOstats to test gene lists for GO term association. Bioinformatics 23: 257–258. 10.1093/bioinformatics/btl567 17098774

[74] Nobile CJ, Nett JE, Hernday AD, Homann OR, Deneault JS, Nantel A, Andes DR, Johnson AD, Mitchell AP (2009) Biofilm Matrix Regulation by *Candida albicans* Zap1. PLoS Biol 7: e1000133 10.1371/journal.pbio.1000133 19529758 PMC2688839

[75] Rupniak HT, Rowlatt C, Lane EB, Steele JG, Trejdosiewicz LK, Laskiewicz B, Povey S, Hill BT (1985) Characteristics of 4 New Human Cell-Lines Derived from Squamous-Cell Carcinomas of the Head and Neck. Journal of the National Cancer Institute 75: 621–635. 2413234

[76] Care RA, Trevethick J, Binley KM, Sudbery PE (1999) The *MET3* promoter: a new tool for *Candida albicans* molecular genetics. Mol Microbiol 34: 792–798. 10.1046/j.1365-2958.1999.01641.x 10564518

[77] Böckmuhl DP, Krishnamurthy S, Gerads M, Sonneborn A, Ernst JF (2001) Distinct and redundant roles of the two protein kinase A isoforms Tpk1p and Tpk2p in morphogenesis and growth of *Candida albicans* . Mol Microbiol 42: 1243–1257. 11886556 10.1046/j.1365-2958.2001.02688.x

[78] Chen JY, Zhou S, Wang Q, Chen X, Pan T, Liu HP (2000) Crk1, a novel Cdc2-related protein kinase, is required for hyphal development and virulence in *Candida albicans* . Mol Cell Biol 20: 8696–8708. 10.1128/MCB.20.23.8696-8708.2000 11073971 PMC86484

[79] Shapiro RS, Sellam A, Tebbji F, Whiteway M, Nantel A, Cowen LE (2012) Pho85, Pcl1, and Hms1 Signaling Governs Candida albicans Morphogenesis Induced by High Temperature or Hsp90 Compromise. Curr Biol 22: 461–470. 10.1016/j.cub.2012.01.062 22365851

[80] Smith DA, Nicholls S, Morgan BA, Brown AJP, Quinn J (2004) A conserved stress-activated protein kinase regulates a core stress response in the human pathogen Candida albicans. Mol Biol Cell 15: 4179–4190. 10.1091/mbc.E04-03-0181 15229284 PMC515350

[81] Csank C, Schroppel K, Leberer E, Harcus D, Mohamed O, Meloche S, Thomas DY, Whiteway M (1998) Roles of the *Candida albicans* mitogen-activated protein kinase homolog, Cek1p, in hyphal development and systemic candidiasis. Infect Immun 66: 2713–2721. 9596738 10.1128/iai.66.6.2713-2721.1998 PMC108260

[82] Goyard S, Knechtle P, Chauvel M, Mallet A, Prevost MC, Proux C, Coppee JY, Schwartz P, Dromer F, Park H, Filler SG, Janbon G, d’Enfert C (2008) The Yak1 kinase is involved in the initiation and maintenance of hyphal growth in *Candida albicans* . Mol Biol Cell 19: 2251–2266. 10.1091/mbc.E07-09-0960 18321992 PMC2366847

