# Supplementary figures and images for: Cell Cycle-Independent Phospho-Regulation of Fkh2 during Hyphal Growth Regulates Candida albicans Pathogenesis
Source: PLoS Pathog. 2015 Jan 24;11(1):e1004630. doi: 10.1371/journal.ppat.1004630 (PMC4305328; doi:10.1371/journal.ppat.1004630)

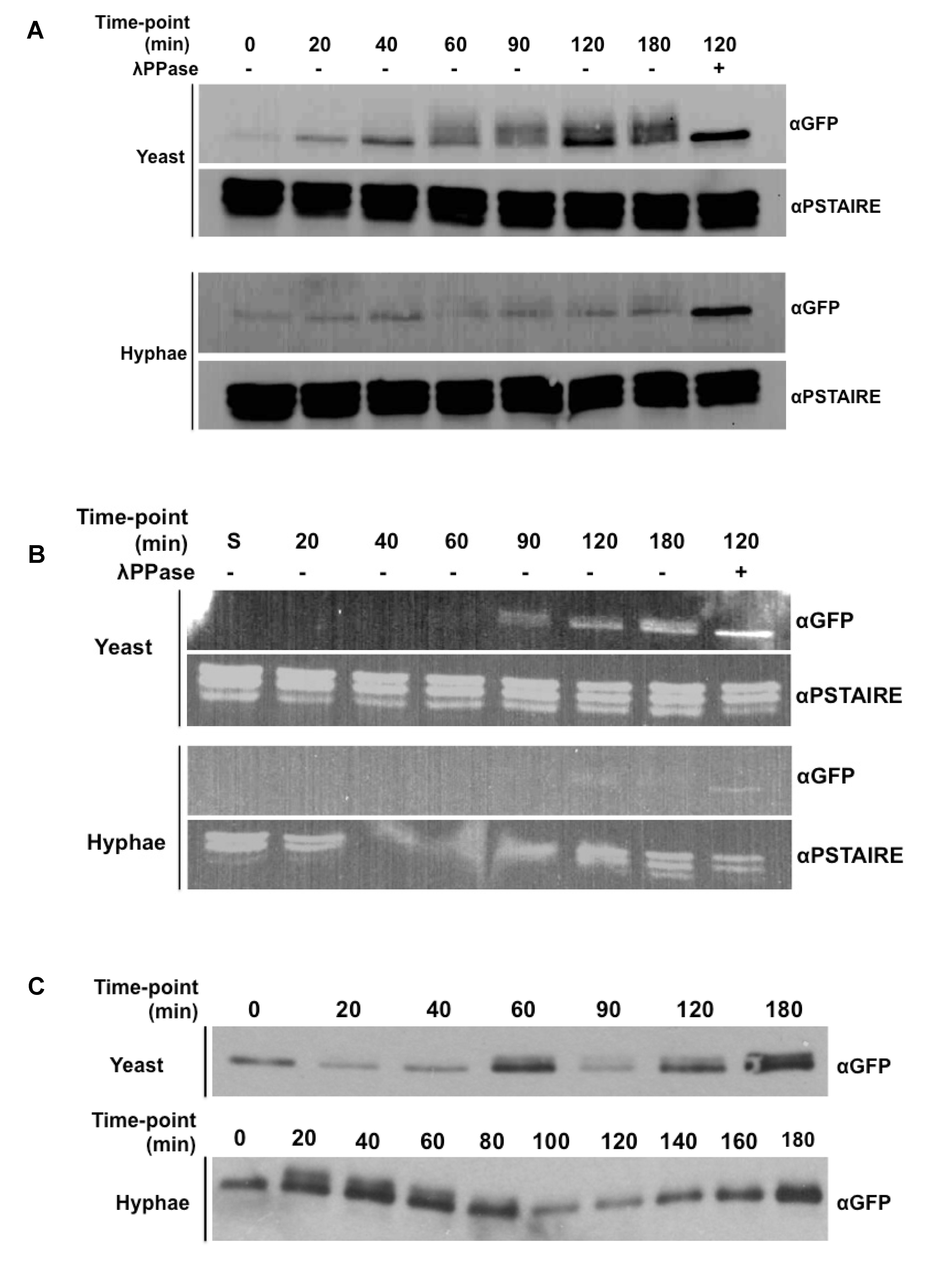

Supplement: S1 Fig — Yeast and hyphal time-courses were carried out for Orf19.3469-GFP (A) Orf19.1948-GFP (B) and Fkh2-YFP (C). 30 μg total protein was run for each time point. In A and B αPSTAIRE was used as a control for equal loading. (TIF) [file ppat.1004630.s001.tif]

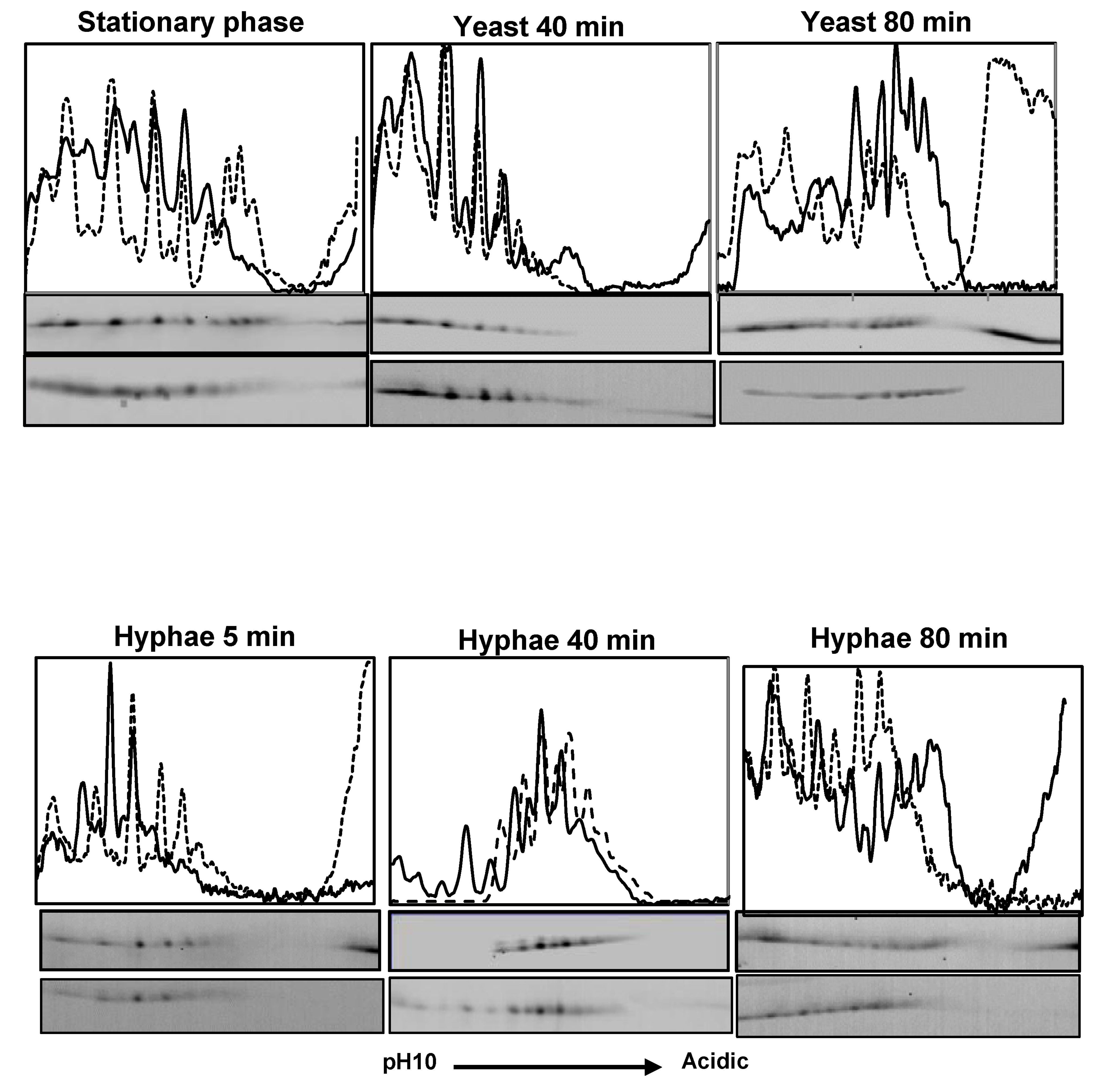

Supplement: S2 Fig — Independent replication of the experiment shown in Fig. 1. Replicate results are shown on the profiles as a solid black line, with the grey dashed line representing the initial profile from Fig. 1. The top panels of gels are those from Fig. 1, with the replicates present in the bottom panel. (TIF) [file ppat.1004630.s002.tif]

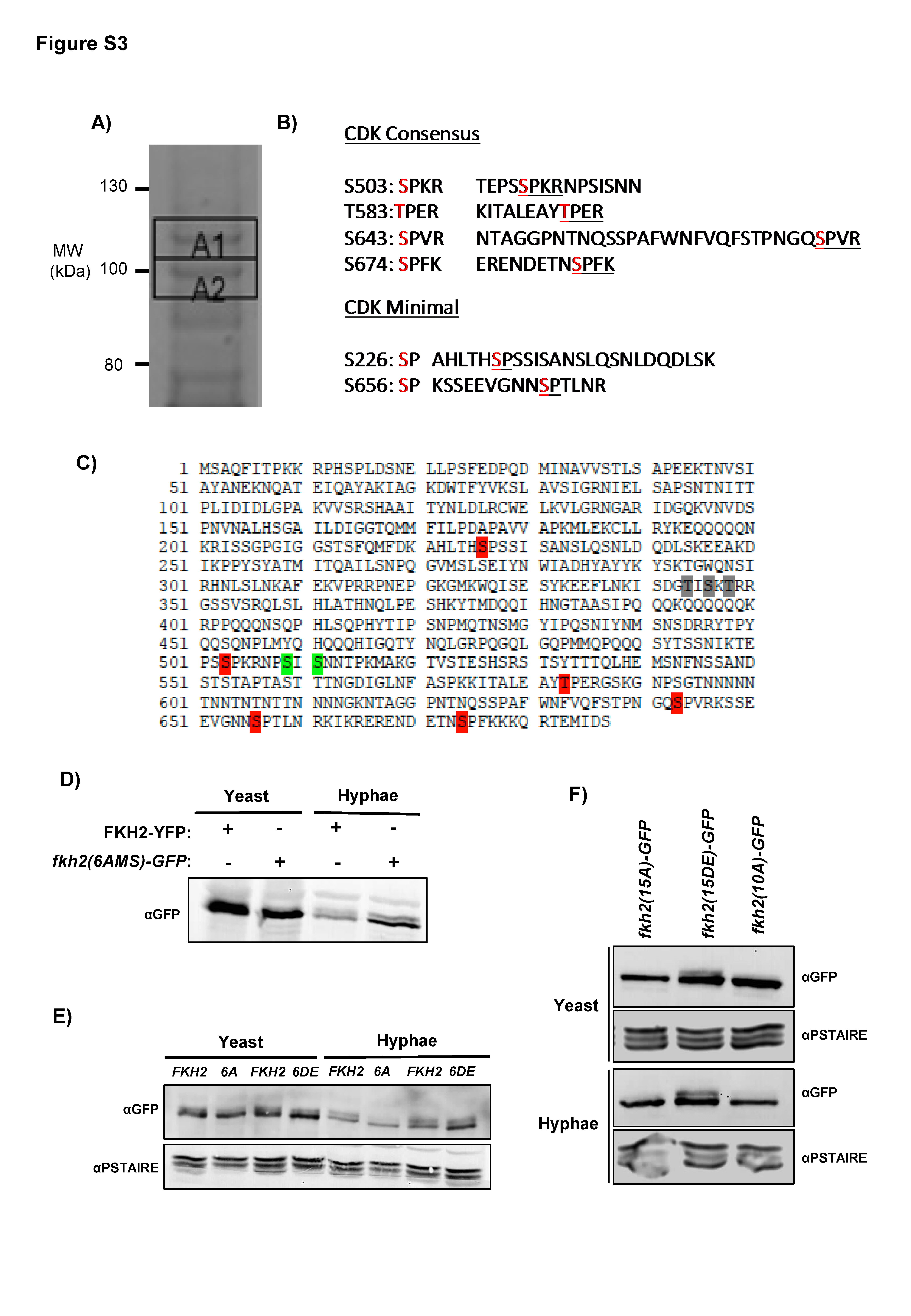

Supplement: S3 Fig — A) Coomassie Blue stained gel of Fkh2-HA purified from 5 L of culture 40 min after hyphae were induced from unbudded stationary phase yeast cells. Bands A1 and A2 indicate those excised for MS analysis. B) List of phospho-peptides detected with phosphorylation occurring in Cdc28 consensus motifs. C) All phospho-sites detected by phospho-peptide mapping. Red—significant hit, Grey—possible phosphorylation site, Green—either site is phosphorylated. D) Mutation of significant hits from phospho-peptide mapping does not affect Fkh2’s associated phospho-shift. FKH2-YFP and fkh2(6AMS)-GFP strains were grown for 80 min in yeast or 40 min hyphal growth conditions before harvesting cells for protein extraction. E) Yeast and hyphal 1D phosphorylation profiles of Cdc28 consensus target site mutants with phosphoacceptor residues mutated to alanine or glutamate/aspartate. F) Mutation of all CDK consensus and minimal sites (15A and 15DE), or all Cdc28 target sites C-terminal to the DNA binding domain in Fkh2 (10A). Strains were grown and samples processed as previously described. (TIF) [file ppat.1004630.s003.tif]

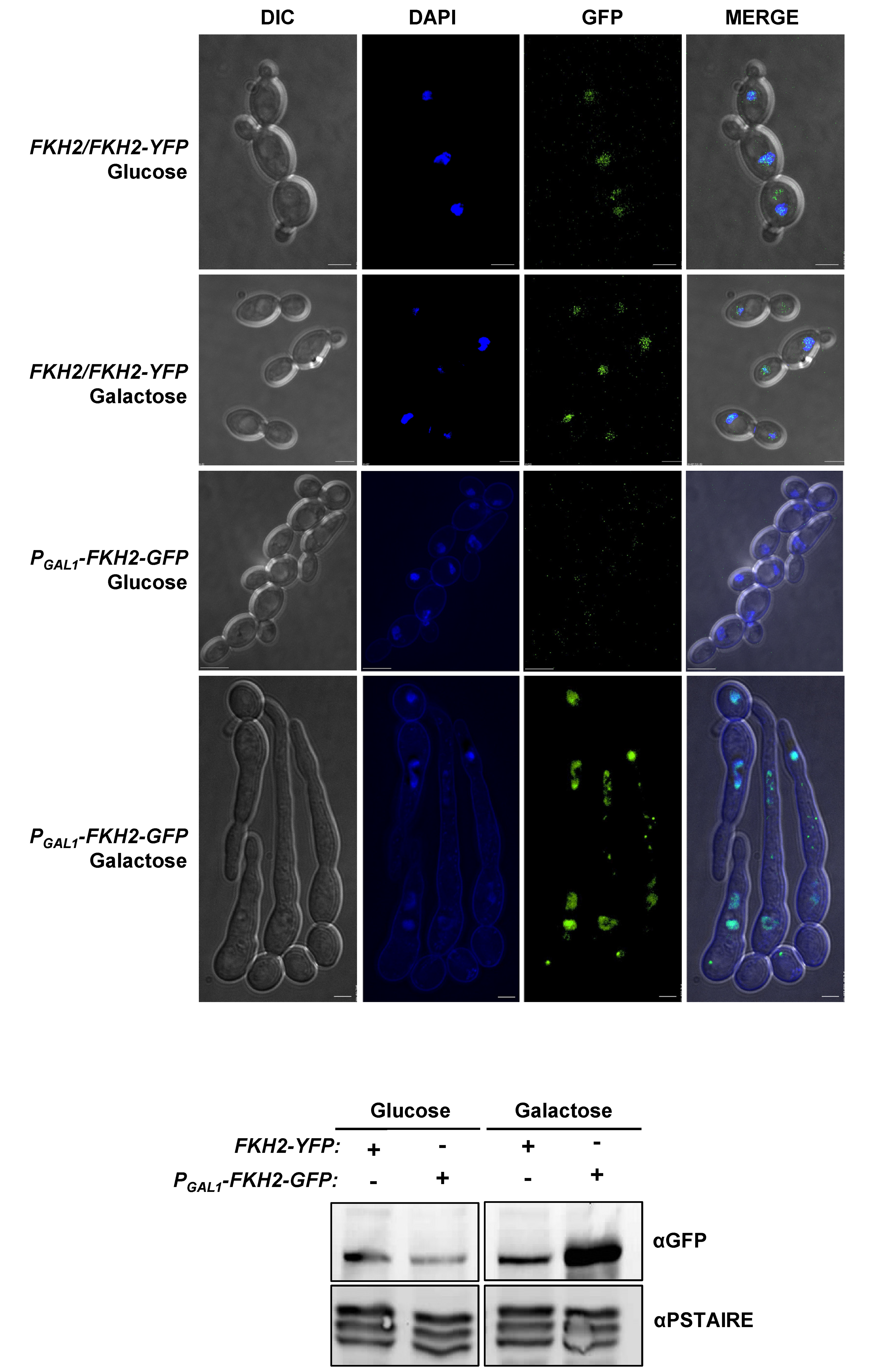

Supplement: S4 Fig — FKH2-YFP and PGAL1-FKH2-GFP were grown to stationary phase in glucose rich media. The strains were then re-inoculated into yeast growth conditions in either glucose (YEPD) or galactose (YEPG) rich media and grown for 3 h at 30°C. A: Cells were washed in PBS, DAPI was added and then GFP/DAPI fluorescence images were taken at x100 magnification. Scale bars represent 10 μm. B: Western blot using a monoclonal antibody to GFP shows that FKH2-GFP was overexpressed from the GAL1 promoter compared to the control FKH2-YFP expressed from its native promoter. (TIF) [file ppat.1004630.s004.tif]

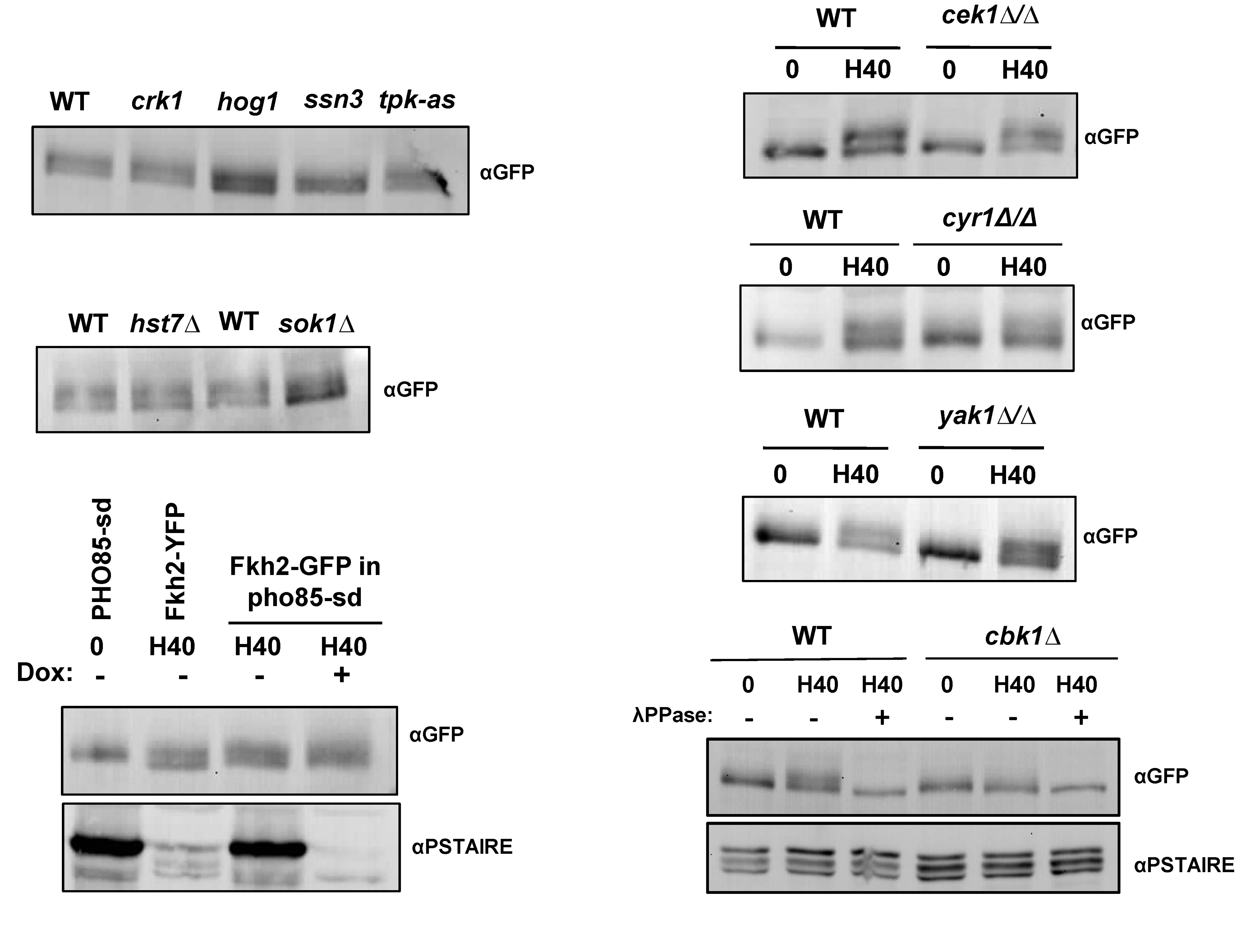

Supplement: S5 Fig — Fkh2 was C-terminally tagged with GFP in a series of kinase mutants generously provided by their original constructors: Tpk1 [77]; Ssn3 [78]; Pho85-sd [79]; Hog1 [80]; Cek1 [81]; Yak1 [82]. Stationary phase yeast cells were induced to form hyphae as described in materials and methods. Panels A,B cells were isolated 40 minutes after hyphal induction and the phosphorylation state compared to the wild type by the presence of a band shift in a Western blot using a monoclonal antibody against GFP. C-G) Western blots were prepared from stationary phase samples (0) and samples prepared 40 minutes after hyphal induction (H40). C) Pho85 expressed from the Tet-off promoter was repressed by the addition of 20µg.ml-1 Doxycyclin. G) Only the cbk1Δ strain failed to show the double band 40 minutes after hyphal induction, being present as the stationary phase or phosphatase treated form. All the other strains showed the same double band as the wild type. (TIF) [file ppat.1004630.s005.tif]
