# Supplementary material for: Cell Cycle-Independent Phospho-Regulation of Fkh2 during Hyphal Growth Regulates Candida albicans Pathogenesis
Source: PLoS Pathog. 2015 Jan 24;11(1):e1004630. doi: 10.1371/journal.ppat.1004630 (PMC4305328; doi:10.1371/journal.ppat.1004630)
Supplement: S1 Table — (DOCX) [file ppat.1004630.s008.docx]

**Strains used in this Study**

| Strain # | Strain | Genotype | Source |
| --- | --- | --- | --- |
|  | BWP17 | *ura3::λimm434/ ura3::λimm434 his1::hisG/ his1::hisG arg4::hisG/arg4::hisG* | Wilson et al. 1999 |
|  | BWP17 *ARG4^+^ URA3^+^* | *ura3::λimm434/ URA3 his1::hisG/ his1::hisG arg4::hisG/ARG4* | This Study |
| 1406 | *FKH2-YFP* | *FKH2/FKH2-YFP:URA3* | This study |
| 1468 | *FKH2-YFP, CDC12-MCherry* | BWP17 *FKH2/FKH2-YFP:URA3, CDC12/CDC12-MCherry:ARG4* | This Study |
| 1433 | FKH2-HA | BWP17 *FKH2/FKH2-HA:URA3* |  |
| 1418 | *fkh2∆/FKH2* | BWP17 *FKH2/fkh2::ARG4* | This study |
| 1419 | *fkh2 ∆/∆ (URA3^+^)* | BWP17 *frt-URA3-P_SAP2_-C.a.FLP-frt::fkh2/fkh2::ARG4* | This study |
| 1423 | *fkh2 ∆/∆ (ura3^-^)* | BWP17 *frt-frt::fkh2/fkh2::ARG4* | This study |
| 1471 | *fkh2/FKH2-YFP* | BWP17 *fkh2::ARG4/FKH2-YFP:URA3* | This study |
| 1457.2 | *FKH2(6AMS)-GFP* | BWP17 *frt::fkh2/fkh2::ARG4/fkh2(S226A, S503A, T583A, S643A, S656A, S674A)-GFP:URA3* | This Study |
| 1449 | *fkh2(6A)-GFP* | BWP17 *frt::fkh2/fkh2::ARG4/fkh2(T7A, S503A, S573A, T583A, S643A, S674A)-GFP:URA3* | This study |
| 1450 | *fkh2(6DE)-GFP* | BWP17 *frt::fkh2/fkh2::ARG4/fkh2(T7E, S503D, S573D, T583E, S643D, S674D)-GFP:URA3* | This Study |
| 1451 | *fkh2(15A)-GFP* | BWP17 *frt::fkh2/fkh2::ARG4/fkh2(T7A, S503A, S573A, T583A, S643A, S674A, S14A, T100A, S150A, S226A, T448A, T514A, S627A, T638A, S656A)-GFP:URA3* | This Study |
| 1452 | *fkh2(15DE)-GFP* | BWP17 *frt::fkh2/fkh2::ARG4/fkh2(T7E, S503D, S573D, T583E, S643D, S674D, S14D, T100E, S150D, S226D, T448E, T514E, S627D, T638E, S656D)-GFP:URA3* | This Study |
| 1464 | *fkh2(11A)-GFP* | BWP17 *frt::fkh2/fkh2::ARG4/fkh2(S503A, S573A, T583A, S643A, S674A, S226A, T448A, T514A, S627A, T638A, S656A)-GFP:URA3* | This Study |
| 1459 | *fkh2(1-426)-GFP* | BWP17 *frt::fkh2/fkh2::ARG4/fkh2(1-426)-GFP:URA3* | This Study |
|  | *cdc28-1as* | BWP17 *cdc28::HIS1/cdc28^F80G^:ARG4* | Sinha et al. 2007 |
| 1408 | *FKH2-GFP in ccd28-1as* | BWP17 *cdc28::HIS1/cdc28^F80G^:ARG4, FKH2/FKH2/FKH2-GFP:URA3* | This study |
| 1424 | *FKH2-GFP in Ccn1 -/-* | BWP17 *HIS1::ccn1/ccn1::ARG4, FKH2/FKH2/FKH2-GFP:URA3* | This study |
| 1425 | *FKH2-GFP in Hgc1 -/-* | BWP17 *HIS1::hgc1/hgc1::ARG4, FKH2/FKH2/FKH2-GFP:URA3* | This study |
|  | *CLN3-sd* | BWP17 *Frt::cln3/ARG4:P_MET3_-6Myc-CLN3* | Zeng et al. 2012 |
| 1446 | *FKH2-GFP in CLN3-sd* | BWP17 *Frt::cln3/ARG4:P_MET3_-6Myc-CLN3, FKH2/FKH2/FKH2-GFP:URA3* | This study |
|  | *crk1Δ/Δ* | BWP17 *HIS1::crk1/crk1::ARG4* | Chen et al. 2002 |
| 1470 | *FKH2-GFP in crk1Δ/Δ* | BWP17 *HIS1::crk1/crk1::ARG4, FKH2/FKH2/FKH2-GFP:URA3* | This study |
|  | *hog1 Δ/Δ* | BWP17 *HIS1::hog1/hog1::ARG4* | Smith et al. 2004. |
| 1473 | *FKH2-GFP in*  *hog1 Δ/Δ* | BWP17 *HIS1::hog1/hog1::ARG4, FKH2/FKH2/FKH2-GFP:URA3* | This study |
|  | *tpk2Δ/tpk1-as* | *ura3Δ::l imm434/ura3Δ::l imm434 tpk1^M167A^/ tpk1Δ::hisG/ tpk2Δ::hisG/ tpk2Δ::hisG* | Bockmühl et al., 2001 |
| 1469 | *FKH2-GFP in tpk2Δ/tpk1-as* | *ura3Δ::l imm434/ura3Δ::l imm434 tpk1^M167A^/ tpk1Δ::hisG/ tpk2Δ::hisG/ tpk2Δ::hisG, FKH2/FKH2/FKH2-GFP:URA3* | This study |
|  | *ssn3∆/∆* | *ssn3Δ::C.m.LEU2/ssn3Δ::C.d.HIS1, his1Δ/his1Δ, arg4Δ/arg4Δ, leu2Δ/leu2Δ, ura3Δ/URA3, iro1Δ/IRO1* | Chen and Noble., 2012 |
| 1474 | *FKH2-GFP in ssn3Δ/Δ* | *ssn3Δ::C.m.LEU2/ssn3Δ::C.d.HIS1, his1Δ/his1Δ, arg4Δ/arg4Δ, leu2Δ/leu2Δ, ura3Δ/URA3, iro1Δ/IRO1, FKH2/FKH2-GFP:ARG4* | This Study |
|  | *PHO85-sd* | *arg4Δ/arg4Δ, leu2Δ/leu2Δ, ura3Δ/URA3, iro1Δ/IRO his∆/his1:: tetR-FRTtetO-PHO85/pho85::C.d.HIS1* | Shapiro et al 2012 |
| 1496 | *FKH2-GFP in PHO85-sd* | *arg4Δ/arg4Δ, leu2Δ/leu2Δ, ura3Δ/URA3, iro1Δ/IRO his∆/his1:: tetR-FRTtetO-PHO85/pho85::C.d.HIS1, FKH2/FKH2-GFP:ARG4* | This Study |
|  | *cek1∆/∆* | CAI4 *cek1::hisG/cek1::hisG* | Csank et al. 1998 |
| 1497 | *FKH2-GFP in cek1∆/∆* | CAI4 *cek1::hisG/cek1::hisG, FKH2/FKH2/FKH2-GFP:URA3* | This Study |
|  | *yak1∆/∆* | BWP17 *yak1::HIS1/yak1::ARG4* | Goyard et al 2008 |
| 1489 | *FKH2-GFP in yak1∆/∆* | BWP17 *yak1::HIS1/yak1::ARG4, FKH2/FKH2/FKH2-GFP:URA3* | This Study |
|  | *cyr1∆/∆* | CAI4 *cdc35::hisG/cdc35::hisG* | Rocha et al. 2001 |
| 1490 | *FKH2-GFP in cyr1∆/∆* | CAI4 *cdc35::hisG/cdc35::hisG, FKH2/FKH2/FKH2-GFP:URA3* |  |
|  | *hst7∆/∆* | BWP17 *hst7::tn7-ARG4/hst7:tn7::URA3* | Blankenship et al. 2010 |
| 1484 | *FKH2-GFP in hst7∆/∆* | BWP17 *hst7::tn7-ARG4/hst7:tn7::URA3, FKh2/FKH2/FKH2-GFP:HIS1* |  |
|  | *sok1∆/∆* | BWP17 *sok1::tn7-ARG4/sok1:tn7::URA3* | Blankenship et al. 2010 |
| 1485 | *FKH2-GFP in sok1∆/∆* | BWP17 *sok1::tn7-ARG4/sok1:tn7::URA3, FKH2/FKH2/FKH2-GFP:HIS1* |  |
|  | *cbk1∆/∆* | BWP17 *cbk1::ARG4/cbk1::HIS1* | This study |
| 1491 | *FKH2-GFP in cbk1∆/∆* | *BWP17 cbk1::ARG4/cbk1::HIS1, FKH2/FKH2/FKH2-GFP:URA3* | This Study |
| 1642 | *Cdc28-HA* | BWP17 CDC28-*HA:URA3/CDC28-HA:ARG4* |  |
| 1498 | *fkh2(S542A)-GFP* | BWP17 *frt::fkh2/fkh2::ARG4/fkh2(S542A)-GFP:URA3* | This Study |
|  | *Mob2-HA* | BWP17 *MOB2-HA:URA3/MOB2-HA:ARG4* |  |
| 1494 | *POB3-HA* | BWP17 *POB3/POB3-HA:ARG4* | This Study |
| 1444 | *POB3-HA, FKH2-YFP* | BWP17 *FKH2/FKH2-YFP:URA3, POB3/POB3-HA:ARG4* | This Study |
| 1493 | *SRP1-HA* | BWP17 *SRP1/SRP1-HA:ARG4* | This Study |
| 1445 | *SRP1-HA, FKH2-YFP* | BWP17 *FKH2/FKH2-YFP:URA3, POB3/POB3-HA:ARG4* | This Study |
| 1481 | *POB3-HA, fkh2∆/FKH2-YFP* | BWP17 *ARG4::fkh2/FKH2-YFP:URA3, POB3/POB3-HA:HIS1* | This Study |
| 1483 | *POB3-HA, fkh2∆/fkh2(6A)-YFP* | BWP17 *frt::fkh2/fkh2::ARG4/fkh2(T7A, S503A, S573A, T583A, S643A, S674A)-GFP:URA3, POB3/POB3-HA:HIS1* | This Study |
| 1482 | *POB3-HA, fkh2∆/fkh2(6DE)-YFP* | BWP17 *frt::fkh2/fkh2::ARG4/fkh2(T7A, S503A, S573A, T583A, S643A, S674A)-GFP:URA3, POB3/POB3-HA:HIS1* | This Study |
| 1472 | *P_GAL1_-FKH2-GFP* | BWP17 *FKH2/FKH2/P_GAL1_-FKH2-GFP:URA3* | This Study |

**Plasmids used in this study**

| Name | Description | Source |
| --- | --- | --- |
| pYFP-*URA3* | Production of a YFP *URA3* cassette for C-terminal YFP fusions | Gerami-Nejad *et al*., 2001 |
| *FKH2-*3xHA in pCIP10U | *kpnI*-*FKH2*(CT)-*XhoI* fragment of *FKH2* cloned into pCIP10U with 3xHA 3’ of *XhoI* cloning site | This Study |
| pBKs *FKH2* URAF Del | *KpnI*-*FKH2*(5’)-*XhoI* and *NotI*-*FKH2*(3’)-*SacII* fragments cloned upstream and downstream respectively of *C. albicans* URA Flipper selectable marker | This Study |
| pBKs *FKH2 ARG4* Del | pBKs *FKH2* URAF Del with *XhoI*-URAF-*NotI* replaced with *XhoI*-*P_ARG4_ARG4*-*NotI* | This Study |
| pCIP10U *P_FKH2_*-*FKH2*-GFP H3ins | pCIP10U *P_FKH2_*-*FKH2*-GFP vector with *HindIII* restriction site in *FKH2* promoter | This Study |
| pCIP10U *P_FKH2_*-*FKH2*(6A_MS_)-GFP | pCIP10U *P_FKH2_*-*FKH2*-GFP H3ins vector with *fkh2* mutations: S226A, S503A, T583A, S643A, S656A, S674A. | This Study |
| pCIP10U *P_FKH2_*-*FKH2*(6A)-GFP | pCIP10U *P_FKH2_*-*FKH2*-GFP H3ins vector with *fkh2* mutations: T7A, S503A, S573A, T583A, S643A, S674A. | This Study |
| pCIP10U *P_FKH2_*-*FKH2*(6DE)-GFP | pCIP10U P_FKH2_-*FKH2*-GFP H3ins vector with *fkh2* mutations: T7E, S503D, S573D, T583E, S643D, S674D. | This Study |
| pCIP10U *P_FKH2_*-*FKH2*(15A)-GFP | pCIP10U *P_FKH2_*-*fkh2*(*6A_CDK_*)-GFP with further *fkh2* mutations: S14A, T100A, S150A, S226A, T448A, T514A, S627A, T638A, S656A. | This Study |
| pCIP10U *P_FKH2_*-*FKH2*(10A)-GFP | pCIP10U *P_FKH2_*-*FKH2*-GFP digested with *kpnI/XhoI* to remove two C-terminal fragments which were replaced with *kpnI/XhoI* digested fragments from pCIP10U *P_FKH2_*-*FKH2*(15A)-GFP | This Study |
| pCIP10U P_FKH2_-*FKH2*(15DE)-GFP | pCIP10U *P_FKH2_*-*FKH2*(*6E*)-GFP with further *fkh2* mutations: S14D, T100E, S150D, S226D, T448E, T514E, S627D, T638E, S656D. | This Study |
| pCIP10U *P_FKH2_*-*FKH2*(1-426)-GFP | *AscI*-*P_FKH2_* _(_*_HindIII_* _ins)_-*FKH2*(1-1278bp)-*XhoI* fragment PCR amplified from pCIP10U *P_FKH2_*-*FKH2*-GFP H3ins and cloned into pCIP10U with GFP 3’ of *XhoI* cloning site. | This Study |
| pCIP10A *P_FKH2_*-*FKH2*-GFP | *NotI*-*URA3*-*MluI* fragment from pCIP10U *P_FKH2_*-*FKH2*-GFP swapped with *NotI-ARG4-Mlu1* | This Study |
| pCIP10H *P_FKH2_*-*FKH2*-GFP | *NotI*-*ARG4*-*MluI* fragment from pCIP10U *P_FKH2_*-*FKH2*-GFP swapped with *NotI-HIS1-Mlu1* | This Study |
| pCIP10U *P_FKH2_*-*fkh2*(S542A)-GFP | pCIP10U *P_FKH2_*-*FKH2*-GFP H3ins vector with *fkh2* mutations: S542A | This Study |
| pCIP10U *P_GAL1_*-*FKH2-GFP* | pCIP10U with *AscI-P_GAL1_-BamHI* fragment cloned upstream of *FKH2-GFP* to replace the native promoter | This Study |
| GST-*FKH2(CT*) | *BamHI*-*FKH2*(CT)-*XhoI* fragment generated by skip PCR from pCIP10U *P_FKH2_*-*FKH2*-GFP to remove intron, cloned into pGEX-4T1 | This Study |
| GST-*fkh2(CT)6A* | *BamHI*-*FKH2*(CT)6A_CDK_-*XhoI* fragment generated by skip PCR from pCIP10U *P_FKH2_*-*fkh2*(6A_CDK_)-GFP to remove intron, cloned into pGEX-4T1 | This Study |
| pCIP10A *CDC12-*MCherry | *kpnI*-*CDC12*(CT)-*XhoI* fragment cloned into pCIP10A M-cherry vector | This Study |
| pCIP10U *P_FKH2_*-*FKH2*-6xMyc | *AscI*-*P_FKH2_*-*FKH2*-*XhoI* fragment cloned into pCIP10U with 6xMyc 3’ of *XhoI* cloning site | This study |
| pCIP10A *POB3-*HA | *KpnI*-*POB3*(CT)-*XhoI* fragment cloned upstream of HA epitope sequence in pCIP10A vector | This Study |
| pCIP10A *SRP1-*HA | *KpnI*-*SRP1*(CT)-*XhoI* fragment cloned upstream of HA epitope sequence in pCIP10A vector | This Study |
| pCIP10H *POB3-*HA | *POB3-*HA in pCIP10A vector with the *Candida* selectable *ARG4* marker swapped to *HIS1* using *MluI/NotI* | This Study |

**Primers used in this study**

| Primer | Description | Sequence |
| --- | --- | --- |
| *FKH2*-URA Rev | 3’ Primer for YFP tagging FKH2 at the C-terminus | CCTCTTATGTATGTAATTATTACGTATATACGTGTTATGTATCGTTCCTAAATCTACCTTTGTCAATGGTCTAGAAGGACCACCTTTGATTG |
| *FKH2*-YFP Fwd | 5’ Primer for YFP tagging Fkh2 at the C-terminus | CGAGAGAGGGAAAATGATGAAACCAATTCGCCATTTAAAAAAAAACAACGGACTGAAATGATTGATCTGGGTGGTGGTTCTAAAGGTGAAGAATTATT |
| *FKH2*-Fwd Check | 5’ Primer to check FKH2 C-terminal YFP tag | GGGCGACCACAAGGCCAGCTAGG |
| *FKH2*-*KpnI* AF | 5’Primer to amplify 5’ region upstream of FKH2 | GGTACCCTTTATCAACCAATAACACAC |
| *FKH2*-*XhoI* BR | 3’ Primer to amplify 5’ region upstream of FKH2 | CTCGAGGTAAATCCTAGCAAAAAAATG |
| *FKH2*-*NotI* CF | 5’Primer to amplify 3’ region downstream of FKH2 | GCGGCCGCCACTTCACTCACACAGATATA |
| *FKH2*-*SacII* DR | 3’ Primer to amplify 3’ region downstream of FKH2 | CCGCGGGGAATTTGATATATCATTGAA |
| T3 | 5’ Sequencing primer upstream of cloning sites in pCIP10 and pBKs | GCAATTAACCCTCACTAAAGG |
| T7 | 3’ Sequencing primer downstream of cloning sites in pCIP10 and pBKs | TAATACGACTCACTATAGGG |
| *FKH2* Del Cdk Fwd | 5’ Primer for checking FKH2 deletions | GAACAAAAAATTAAGACAGAAC |
| URAF Del Rev Chk | 3’ primer for checking URA flipper deletions | CGGAAATCTATTGTTGTTGTCAC |
| *ARG4* Del Rev Chk | 3’ primer for checking ARG4 cassette deletions | GAAATGACTGAATTATGTCGGTC |
| *FKH2*+5’ *AscI* Fwd | 5’ Primer to amplify FKH2 and 600bp of its promoter | GGCGCGCCCAGCACTTTTGATCATTCACG |
| *FKH2* *XhoI* Rev | 3’ Primer to amplify FKH2 | CTCGAGCAGATCAATCATTTCAGTCCG |
| *FKH2* Fwd Seq 1 | FKH2 sequencing primer, binds in promoter | CAA CCA ATA ACA CAC TTT TGA TTC |
| *FKH2* Fwd Seq 2 | FKH2 sequencing primer | CAG GAT ATG ATA AAT GCA GTG G |
| *FKH2* Fwd Seq 3 | FKH2 sequencing primer | GAA CAA ACG AAT TTC AAG TGG |
| *FKH2* Fwd Seq 4 | FKH2 sequencing primer | CTC AGC AAC AAA AAC AAC AAC |
| *FKH2* (Pro) *HindIII* insertion | Mutagenic primer for HindIII mutation in FKH2 Promoter | AATTGGAAGCAAAAGTAAGAA AAGCTT TCTTTATCCTGTTTTTTTTTA |
| *FKH2* T7A | Mutagenic primer to mutate T7 to A in Fkh2 | ATG TCA GCA CAA TTT ATC GCA CCG AAA AAG CGT CCC |
| *FKH2* T7E | Mutagenic primer to mutate T7 to E in Fkh2 | CGA TTA TTT ACA AAT GTC AGC ACA ATT TAT CGA GCC GAA AAA GCG TCC CCA CTC AC |
| *FKH2* S14A | Mutagenic primer to mutate T14 to A in Fkh2 | CCGAAAAAGCGTCCCCACGCACCACTAGATAGTAATGAATTACTTC |
| *FKH2* S14D | Mutagenic primer to mutate T14 to D in Fkh2 | CCGAAAAAGCGTCCCCACGATCCACTAGATAGTAATGAATTACTTC |
| *FKH2* T100A | Mutagenic primer to mutate T100 to A in Fkh2 | CCAGTAATACTAATATAACTGCACCTTTAATAGATATTGACTTGGG |
| *FKH2* T100E | Mutagenic primer to mutate T100 to E in Fkh2 | CCAGTAATACTAATATAACTGAACCTTTAATAGATATTGACTTGGG |
| *FKH2* S150A | Mutagenic primer to mutate S150 to A in Fkh2 | GTCAAAAAGTTAATGTTGATGCACCAAATGTTAATGCATTACATTC |
| *FKH2* S150D | Mutagenic primer to mutate S150 to D in Fkh2 | GTCAAAAAGTTAATGTTGATGATCCAAATGTTAATGCATTACATTC |
| *FKH2* S226A | Mutagenic primer to mutate S226 to A in Fkh2 | GATAAAGCTCATCTAACTCATGCCCCTTCATCTATTTCAGCAAACTCG |
| *FKH2* S226D | Mutagenic primer to mutate S226 to D in Fkh2 | GATAAAGCTCATCTAACTCATGACCCTTCATCTATTTCAGCAAACTCG |
| *FKH2* T448A | Mutagenic primer to mutate T448 to A in Fkh2 | CAAATAGCGATCGTCGTTATGCACCATACCAACAACTGCAAAACCC |
| *FKH2* T448E | Mutagenic primer to mutate T448 to E in Fkh2 | CAAATAGCGATCGTCGTTATGAACCATACCAACAACTGCAAAACCC |
| *FKH2* S503A | Mutagenic primer to mutate S503 to A in Fkh2 | CAT TAA AAC CGA GCC CAG TGC TCC AAA AAG AAA TCCA TCT |
| *FKH2* S503D | Mutagenic primer to mutate S503 to D in Fkh2 | CTG AGT AAC ATT AAA ACC GAG CCC AGT GAT CCA AAA AGA AAT CCA TCT A |
| *FKH2* T514A | Mutagenic primer to mutate T514 to A in Fkh2 | ATCCATCTATTTCTAACAACGCACCAAAGATGGCTAAAGGCACAGG |
| *FKH2* T514E | Mutagenic primer to mutate T514 to E in Fkh2 | ATCCATCTATTTCTAACAACGAACCAAAGATGGCTAAAGGCACAGG |
| *FKH2* S572A | Mutagenic primer to mutate S572 to A in Fkh2 | GAG ACA TTG GAT TAA ACT TTG CCG CTC CCA AAA AGA TAA CTG CTT TAG |
| *FKH2* S572D | Mutagenic primer to mutate S572 to D in Fkh2 | GGA GAC ATT GGA TTA AAC TTT GCC GAT CCC AAA AAG ATA ACT GCT TTA GA |
| *FKH2* T583A | Mutagenic primer to mutate T583 to A in Fkh2 | AGA TAA CTG CTT TAG AAG CCT ATG CGC CGG AAA GAG |
| *FKH2* T583E | Mutagenic primer to mutate T583 to E in Fkh2 | CCA AAA AGA TAA CTG CTT TAG AAG CCT ATG AGC CGG AAA GAG GTT C |
| *FKH2* S627A and T638A | Mutagenic primer to mutate S627 and T638 to A in Fkh2 | CCAAATACAAATCAATCGGCACCGGCATTTTGGAATTTTGTTCAATTTAGTGCACCTAATGGACAA |
| *FKH2* S627D and T638E | Mutagenic primer to mutate S627 and T638 to D and E respecitvely in Fkh2 | CCAAATACAAATCAATCGGATCCGGCATTTTGGAATTTTGTTCAATTTAGTGAACCTAATGGACAA |
| *FKH2* S643A | Mutagenic primer to mutate S643 to A in Fkh2 | TTT GTT CAA TTT AGT ACA CCT AAT GGA CAA GCA CCA GTA AGA AAA AG |
| *FKH2* S643D | Mutagenic primer to mutate S643 to D in Fkh2 | TTT GTT CAA TTT AGT ACA CCT AAT GGA CAA GAT CCA GTA AGA AAA AGT AGT GAA GAA GTA |
| *FKH2* S656A | Mutagenic primer to mutate S656 to A in Fkh2 | GTGAAGAAGTAGGGAATAATGCTCCTACATTGAATAGAAAAATAAA |
| *FKH2* S656D | Mutagenic primer to mutate S656 to D in Fkh2 | GTGAAGAAGTAGGGAATAATGATCCTACATTGAATAGAAAAATAAA |
| *FKH2* S674A | Mutagenic primer to mutate S674 to A in Fkh2 | AGC GAG AGA GGG AAA ATG ATG AAA CCA ATG CGC CAT TTA AAA AAA |
| *FKH2* S674D | Mutagenic primer to mutate S674 to D in Fkh2 | AAA AAT AAA GCG AGA GAG GGA AAA TGA TGA AAC CAA TGA TCC ATT TAA AAA AAA ACA ACG GAC |
| *FKH2 S542A* | Mutagenic primer to mutate S542 to A in Fkh2 | AGAAAGCCATTCGAGAAGCACAGCGTATACGACAACACAGTTGCACG |
| *FKH2*(1-426) *XhoI* Rev | Reverse primer to truncate Fkh2 to residue 426 | CCG CTCGAG GGTTTGCATGGGATTAGAAGG |
| *P_FKH2_* *BamHI* insertion | Mutagenic primer to introduce BamHI restriction site upstream of FKH2 in vector for promoter swap | TTTTAGTCATTTTTTTGCTA GGAT CC ACGATTATTTACAAATGTCA |
| *AscI*-*P_GAL1_* Fwd | 5’ Primer to amplify the *GAL1* promoter | TTGGCGCGCCGTTTAAGTTTTTATTATTATGAGTTG |
| *BamHI*-*P_GAL1_* Rev | 3’ Primer to amplify the *GAL1* promoter | CGCGGATCCGGTATAACTCTTTCTTATAAAAATCG |
| *POB3*(CT)-*KpnI* Fwd | 5’ primer to amplify Pob3(CT) | CGG GGTACC GCAGTAGTCAACGAAACTAGTGC |
| *POB3*(CT)-*XhoI* Rev | 3’ primer to amplify Pob3(CT) | CCG CTCGAG ATTCTTGGCCTTTTTCTTAGG |
| SRP1(CT)-KpnI Fwd | 5’ primer to amplify Srp1(CT) | CGG GGTACC GTGACTCTTCTGACAATCGAGAC |
| SRP1(CT)-XhoI Rev | 3’ primer to amplify Srp1(CT) | CCG CTCGAG AAACTGAAAGTTCTGTTGTTGTTG |
| FKH2(CT)A Fwd-BamHI | 5’ primer for FKH2(CT) skip PCR upstream fragment | CGC GGATCC CCTTCTAATCCCATGCAAACC |
| FKH2(CT)B Rev | 3’ primer for FKH2(CT) skip PCR upstream fragment with overlap | TCTGTTGATACTGTGCCTTTAGCCATCTTTG |
| FKH2(CT)C Fwd | 5’ primer for FKH2(CT) skip PCR downstream fragment | AAAGGCACAGTATCAACAGAAAGCCATTCGAG |
| ADE2q-F1 | 5’ Primer for amplification of *ADE2* in qPCR reactions | AAGGAATCTCCATTGGTGGG |
| ADE2q-R1 | 3’ Primer for amplification of *ADE2* in qPCR reactions | GGCCGCCACCATACCTGGCA |
| HGC1q-F1 | 5’ Primer for amplification of *HGC1* in qPCR reactions | AATATGCAACCACCACCACC |
| HGC1q-R1 | 3’ Primer for the amplification of *HGC1* in qPCR reactions | GAAACAGCACGAGAACCAGC |
| KIP4q-F1 | 5’ Primer for amplification of *KIP4* in qPCR reactions | TACTGGGTTTGGAGGTGGAC |
| KIP4q-R1 | 3’ Primer for amplification of *KIP4* in qPCR reactions | TTGCTGTTGTTGATGCTGCT |
| SAP4q-F1 | 5’ Primer for amplification of *SAP4* in qPCR reactions | TGGTGGTATTGACAAGGCCA |
| SAP4q-R1 | 3’ Primer for amplification of *SAP4* in qPCR reactions | ACACCAGCGTTGACATTGAC |
| ECE1q-F1 | 5’ Primer for amplification of *ECE1* in qPCR reactions | TCTCAAGCTGCCATCATCC |
| ECE1q-R1 | 3’ Primer for amplification of *ECE1* in qPCR reactions | TTGTGGAATGTTGCCAAGAA |
